# A study of CCD8 genes/proteins in seven monocots and eight dicots

**DOI:** 10.1371/journal.pone.0213531

**Published:** 2019-03-12

**Authors:** Ritu Batra, Priyanka Agarwal, Sandhya Tyagi, Dinesh Kumar Saini, Vikas Kumar, Anuj Kumar, Sanjay Kumar, Harindra Singh Balyan, Renu Pandey, Pushpendra Kumar Gupta

**Affiliations:** 1 Department of Genetics and Plant Breeding, CCS University, Meerut, India; 2 Division of Plant Physiology, ICAR-Indian Agricultural Research Institute, New Delhi, India; 3 Advance Center for Computational & Applied Biotechnology, Uttarakhand Council for Biotechnology (UCB), Dehradun, India; 4 Bioinformatics Centre, Biotech Park, Lucknow, India; Huazhong University of Science and Technology, CHINA

## Abstract

In plants, the enzyme CCD8 (carotenoid cleavage dioxygenase 8) is involved in the synthesis of an important hormone, strigolactone, and therefore, plays an important role in controlling growth and development. Using cDNA and protein sequence derived from the gene *ZmCCD8* from maize, we identified putative orthologs of the gene encoding CCD8 in six other monocots and eight dicots; the sequence similarity ranged from 52–75.9% at the gene level and 60.9–93.7% at the protein level. The average length of the gene was ~3.3 kb (range: 2.08 to 3.98 kb), although the number of introns within the genes differed (4 or 5 in dicots and 3 or 4 in monocots, except in *T*. *urartu* with 6 introns). Several cis-acting regulatory elements were identified in the promoters of CCD8 genes, which are known to respond to biotic and abiotic stresses. The N-terminal end (up to ~70 amino acids) of CCD8 proteins was highly variable due to insertions, deletions and mismatches. The variation in genes and proteins were particularly conspicuous in *T*. *urartu* and *Ae*. *tauschii* among the monocots and *A*. *thaliana* and *P*. *persica* among the dicots. In CCD8 proteins, 12 motifs were also identified, of which 6 were novel; 4 of these novel motifs occurred in all the 15 species. The 3D structures of proteins had the characteristic features of the related enzyme apocarotenoid oxygenase (ACO) of *Synechocystis* (a representative of cyanobacteria). The results of qRT-PCR in wheat revealed that under phosphorous (P)-starved condition (relative to expression under optimum P used as control), the expression of TaCCD8 genes increased ~37 fold in root tissue of the cultivar C306 and ~33 fold in shoot tissue of the cultivar HUW468 (the two cultivars differed in their P-use efficiency). This suggested that expression of TaCCD8 genes is genotype-dependent and tissue-specific and is regulated under different levels of P supply.

## Introduction

In plants, CCD8 (carotenoid cleavage dioxygenase 8) is an important enzyme belonging to the family of CCD enzymes. They derive their name from the fact that the CCD-mediated reactions involve cleavage of carotenoids, involving use of dioxygen or molecular oxygen (O_2_). The cleavage products in most cases are apocarotenoids, which carry each an aldehyde or ketone group at the cleavage site. CCDs are further classified into CCD1, CCD2, CCD4, CCD7 and CCD8 on the basis of cleavage position and/or their substrate preferences [1,2]. In different plant species, the CCD8 is also known by the following different names: MAX4 (Arabidopsis), RMS1 (pea), D10 (rice), and DAD1 (petunia) [1,2].

**Fig 1:**
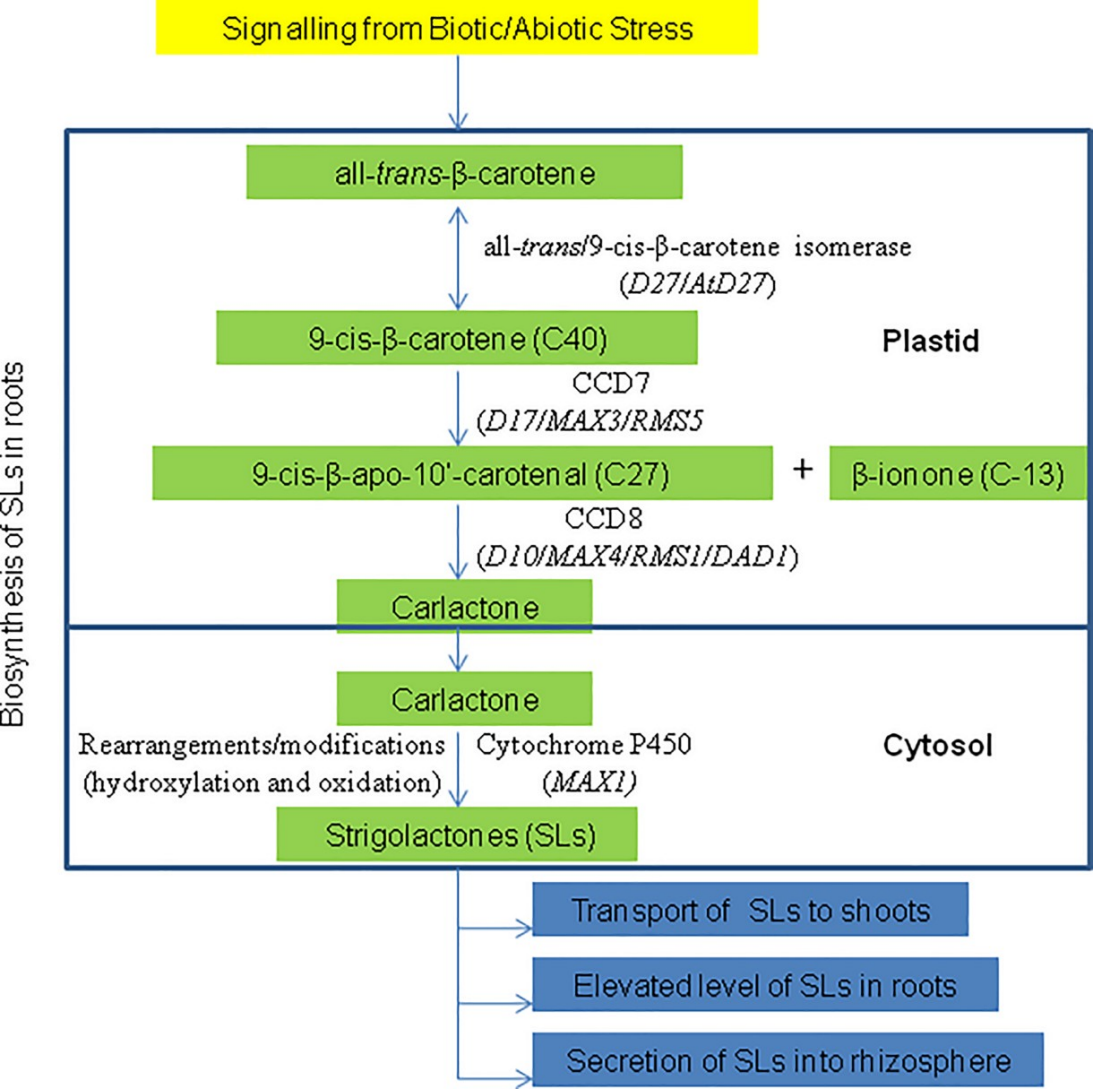
Pathway for the biosynthesis of strigolactone. *D*, dwarf; *AtD*, *Arabidopsis thaliana* dwarf; *MAX*, more axillary growth; *RMS*, ramosus; *DAD*, decreased apical dominance [3–5].

Among all the CCDs, CCD7 and CCD8 received major attention, since these are involved in the catalysis of two consecutive upstream steps of the biosynthesis of strigolactones (SLs), which are important plant hormones with diverse functions including their role as signaling molecules. Through a series of reactions including *cis*-*trans* isomerization, CCD8 is involved in the production of carlactone (Fig 1) which serves as a substrate for P_450_ enzymes, leading to the production of different forms of SLs [3–5]. Since the expression level of CCD8 directly determines the level of the synthesis of SLs, it is believed to be the critical enzyme in SL biosynthesis [6]. The CCD8 genes are also subjected to a feedback regulation [7], because the mutants exhibiting low concentration of SLs, have been shown to experience an upregulation of the expression of CCD8 in several plant systems including pea, petunia, kiwi fruit and the moss *Physcomitrella patens* [8–10]. CCD8 and SLs have also been shown to respond to abiotic stresses, particularly involving the conditions of low inorganic phosphate (Pi) [5,11].

The enzyme CCD8 is largely produced in the roots in a tissue-specific manner, although the gene is also known to be expressed in the shoot and inflorescence [12]. In the roots, the enzyme takes part in the synthesis of SLs, which at higher concentration modulate root system architecture (RSA). SLs are also secreted from the roots into the rhizosphere and exhibit interactions with arbuscular mycorrhizal fungi (AMF), which promote the absorption of nutrients by plants from the soil [13,14]. These are also transported from the roots to the aerial parts of the plant [1,5,15]. The CCD8 (through synthesis of SLs) also controls shoot architecture by inhibiting axillary bud growth and responds to abiotic stresses including low Pi in the soil [5,16,17]. Therefore, CCD8 knock-out mutants showing enhanced branching may be used for improving plant architecture for increased productivity[18–22].

The phylogenetic analysis of CCD8 genes from a number of monocots and dicots has also been conducted, suggesting conserved nature of these genes [23,24]. However, the earlier studies involving characterization of CCD8 genes cover only a few plant systems, including Arabidopsis (MAX4), pea (RMS1), rice (D10), petunia (DAD1), tomato, potato, and maize [6,17,19,24–27]. Therefore, the variation in the structure of these genes and the corresponding proteins in a wide spectrum of monocots and dicots still remains to be worked out.

The present report describes the structure of CCD8 genes and the corresponding proteins in 15 species, which include seven monocots (including maize) and eight dicots. *In silico* expression of CCD8 genes in seven of the above 15 species was also examined in different cells/tissues, at different developmental stages and under conditions of limited supply of phosphate. Using wet-lab experiments involving qRT-PCR, expression levels of TaCCD8 genes was also examined under different conditions of P supply in two wheat cultivars (C306 and HUW468), which are known to differ in their response to abiotic stresses. Evolutionary relationships among these CCD8 genes were inferred using CCD8 proteins. The 3D structures of the CCD8 proteins belonging to all the 15 species were studied for the first time, which will be helpful in the detailed analysis of their functions. The detailed structure of CCD8 genes resolved during the present study will help in the study of allelic variation of the CCD8 genes in monocots and dicots. This should prove useful in planning strategies to improve the plant architecture for enhanced productivity and for designing genotypes suitable for low input conditions.

## Materials and methods

### Identification of ‘putative’ orthologs

Full-length cDNA and protein sequence of *Z*. *mays* CCD8 were available in the database and were used as a reference in tBLASTx and tBLASTn analysis. Thus, ‘putative’ orthologs for the genes encoding CCD8 were identified in 14 other species of higher plants including six monocots (*Triticum aestivum*, *Triticum urartu*, *Aegilops tauschii*, *Oryza sativa*, *Brachypodium distachyon* and *Sorghum bicolor*) and eight dicots (*Arabidopsis thaliana*, *Glycine max*, *Vitis vinifera*, *Solanum lycopersicum*, *Theobroma cacao*, *Populus trichocarpa*, *Prunus persica* and *Medicago truncatula*). The methods used for the identification of putative orthologs were described earlier [28–30]. Briefly, the following criteria were used for the identification of ‘putative’ orthologs: (*i*) high level (>60%) of sequence similarity and high query coverage along the protein length; (*ii*) presence of all domains and motifs available in the original query sequence; (*iii*) conservation of the relative size and sequence among motifs and domains of the query sequence. The orthologous sequences thus obtained were used to identify full length gene sequences from Ensembl Plants (http://plants.ensembl.org/index.html).

### Analysis of gene structure, synteny and collinearity

Intron-exon junctions in the full length gene sequences were determined using the genomic and coding DNA sequences (CDS) for different species. Intron phases (phase 0,1,2) were identified following criteria used by us earlier [29]. Ka/Ks values defining the ratio of non-synonymous to synonymous substitutions were calculated using MEGA version 6.0.6 (dated April 2015) employing Juke Cantor substitution model [31].

The gene sequences were also evaluated for the presence of simple sequence repeats (SSRs) and retro-elements. For this purpose, repeatmasker version 4.0.5 (http://www.repeatmasker.org/), with default parameters was used. One kb genomic regions upstream of the translation start site (ATG) were evaluated for the presence of cis-regulatory response elements in the promoter regions, using PlantCARE database [32]. Only the response elements on the sense strand showing a matrix value of ≥5 were accepted, following the criteria used by us earlier [29].

Using blocks of 31 genes including CCD8 gene of maize (15 genes flanking each of the two borders of CCD8 gene), synteny and collinearity of the orthologs were studied using the online tool Genomicus [33].

### Protein analyses

#### Multiple sequence alignment

The consensus amino acid sequence was generated through multiple sequence alignment of amino acid sequences belonging to all the orthologs through Geneious software ver 6.6.1 with default settings (http://www.geneious.com). The criteria used for this purpose are described elsewhere [34]. For protein sequence similarity, amino acids at different positions of CCD8 protein in a particular species were compared with the consensus sequence. In monocots, a similarity scale of 1–9 (a separate scale for each of the three wheat homoeologues), and in dicots, a similarity scale of 1–8 was used. A value of zero indicated complete lack of similarity with the consensus, while a value of 9 in monocots and 8 in dicots suggested conservation of amino acids in all the species. Conserved domain in the consensus protein sequence was identified through CDD analysis (http://www.ncbi.nlm.nih.gov/Structure/cdd/wrpsb.cgi).

#### Physico-chemical properties

The ProtParam tool (http://web.expasy.org/protparam/) of ExPASy was used to compute the following physico-chemical properties: (i) amino acid composition (%), (ii) molecular weight, (iii) theoretical isoelectric point (pI), (iv) number of positively/negatively charged residues, (v) instability index, (vi) aliphatic index, and (vii) Grand Average of Hydropathy (GRAVY). Similarly, properties at the secondary level were computed using SOPMA = Self Optimized Prediction Method with Alignment (http://npsapbil.ibcp.fr/cgibinnpsaautomat.pl?page=/NPSA/npsa_sopma.html) tool of NPS (Network Protein Sequence Analysis). Motif search was performed using MEME suite (http://meme-suite.org/) using the following parameters: (*i*) maximum number of motifs being 50; (*ii*) optimum width of the motif being 50; and (*iii*) the site being 2–600. The sub-cellular localization was detrmined using ProtComp (http://www.softberry.com/berry.phtml?topic=protcomppl&group=programs&subgroup=proloc).

#### 3D structures

The 3D structures were generated employing Swiss Model using as template the 3D X-ray crystallographic structure of a mutant apocarotenoid oxygenase (ACO) of *Synechocystis* (PDB id: 5kk0.1.A). 3D structures thus generated were verified by both geometric and energetic means using the following servers: (*a*) Structure Analysis and Verification Server (SAVES) (http://nihserver.mbi.ucla.edu/SAVES), and (*b*) Swiss-Model server using structure assessment tool. SAVES employed the following software: (*i*) PROCHECK to find out the relative proportion of amino acids, which fall in favoured region, relative to other regions [35]; (*ii*) VERIFY3D to determine the compatibility of an atomic model (3D) with its own amino acid sequence [36] and (*iii*) ERRAT to analyse the statistics of non-bonded interactions between different atom types [37].

### Molecular dynamics simulation, superimposition, ligand binding site analysis and functional annotation of 3D structures

Initially, structure topology files for 3D protein model were prepared by applying Optimized Potential for Liquid Simulations (OPLS) all atom force fields [38,39]. Each protein model was solvated by adding SPC water atoms in cubic periodic box. Ions were added using Genion tool. Energy minimizations were done by applying 1000 steps of steepest descent minimization algorithm followed by 50000 steps of conjugate gradient to minimum energy. Two equilibrium minimizations (NVT and NPT) were done by applying 50000 steps leap-frog integrator for 100ps. Four steps of LINCS algorithm [40] were applied for constrain bond parameter minimizations; Partial Mesh Ewald [41] method was applied for long range electrostatics minimization under 300K temperature. For pressure coupling, the method of Parrinello-Rahman [42] was used, where a Langevin thermostat was used for temperature control. The periodic boundary conditions and SHAKE algorithm [43] were applied for each system for 10 ns of molecular dynamic simulation.

The compactness of the predicted 3D models during MD simulation analysis was examined using radius of gyration (Rg) of each protein, assuming that relatively steady value of Rg would suggest stably folded proteins; Rg value that changes over time was taken to suggest an unfolded protein.

FATCAT server [44] was used to confirm the 3D structures by superimposing the energy minimized 3D structure of CCD8 for each plant species on the 3D structure of maize CCD8. Using energy minimized 3D structures, COACH metaserver (http://zhanglab.ccmb.med.umich.edu/COACH/) and ProFunc server (http://www.ebi.ac.uk/thornton-srv/databases/profunc/) were used to predict the ligand binding sites and to perform functional annotation, respectively.

### Phylogenetic analysis

Phylogenetic analysis was based on amino acid sequences of CCD8 proteins and was undertaken using MEGA version 6 [45]. Phylogenetic tree was constructed using maximum likelihood method, with a bootstrap involving 1000 iterations.

### *In silico* expression

Using microarray data in Genevestigator platform, *in silico* expression analysis was conducted for the CCD8 orthologs of the following seven species: *Z*. *mays*, *T*. *aestivum*, *O*. *sativa*, *B*. *distachyon*, *A*. *thaliana*, *G*. *max*, and *M*. *truncatula*. The expression analysis was conducted in different tissues, at different developmental stages and under varying levels of phosphate.

### qRT-PCR analysis of TaCCD8 genes

#### Plant growing conditions

Seeds of two wheat cvs., namely C306 and HUW468 (which are known to differ for their response to abiotic stresses) were surface sterlized with 0.1% HgCl_2_ for 2 min followed by 5–6 washings with distilled water. The seedlings were raised in hydroponics under controlled conditions at National Phytotron Facility, ICAR-IARI, New Delhi following the method described earlier [46]. Five days after germination, the seedlings were transferred to plastic containers (10 L capacity) in Hoagland solution with low (5 μM) and optimum (500 μM) phosphorous (P) concentrations. The nutrient solution was changed every third day and pH of the nutrient solution was maintained at 5.6 throughout the experiment. Fresh root and shoot tissue samples of the control (optimum P; 500 μM) and treatments were collected on 21^st^, 24^th^ and 25^th^ days in the following order: (i) on 21^st^ day from seedlings grown in low P (LP; 5 μM); (ii) on 24^th^ day from seedlings that were completely starved of P for 3 days after 21 days in low P (P starvation = PS; 0 μM); (iii) on 25^th^ day from seedlings after optimum P was restored (PR; 500 μM) for one day after 24 days. All experiments were carried out in two replications.

#### RNA isolation, cDNA synthesis, primer design and qRT-PCR analysis

Total RNA was isolated from root and shoot tissues using a TRI reagent (Sigma) followed by RNase-free DNase I (Qiagen) treatment for removal of DNA contamination. Reverse transcription reactions were performed using 2.0 μg of total RNA and M-MuLV Reverse Transcriptase (Promega) according to the manufacturer’s instructions.

A common set of primers for the three wheat TaCCD8 genes (located on chromosomes 3A, 3B and 3D) was designed using Primer3 software (forward primer: GCAGCCTCTCGCGGCTG; reverse primer: TCTGTGACGGCGGCAGC). qRT-PCR was performed with PikoReal Real-Time PCR Systems (Thermo Scientific) using PowerUp SYBR Green Master Mix (Applied Biosystems) in two biological replicates each containing three technical replicates. Following cycles of reactions were performed under the following conditions: 95°C for 30 sec, 40 cycles involving 95°C for 5 sec, and 60°C for 34 sec. Constitutive expression of *TaAct2* gene of wheat was used as an endogenous control. The transcript abundance for each gene was normalized with the internal control. The 2^−ΔΔ^Ct values [fold change in gene expression under low P (LP), P starvation (PS) and P replete (PR) conditions vs. the control] were calculated as follows: 2^−ΔΔ^Ct = [(Ct LP/PS/PR test–Ct LP/PS/PR *TaAct*) − (Ct cont test–Ct cont *TaAct*)] [47]. Water in place of cDNA was used as the negative control.

## Results and discussion

### CCD8 genes in 15 species

The details of CCD8 genes for the 15 species (including *Z*. *mays*) are presented in Table 1. In each species examined, a single ortholog for CCD8 was found except in case of wheat, where three orthologs were found, one each for the three sub-genomes (A, B and D sub-genomes).

**Table 1:** Details of CCD8 genes and corresponding cDNA and CDS in 15 selected species.

Species	Ensembl Plants Id's	Gene		cDNA		CDS	
		Length in bp (chr. no.)	Similarity (%)	Length in bp	Similarity (%)	Length in bp	Similarity (%)
**Monocots**							
*Z*. *mays*	Zm00001d043442_T001	3522 (3)	100	2191	100	1719	100
*T*. *aestivum* -A sub-genome	TraesCS3A02G274300	3732 (3)	65.98	2196	78.44	1683	87.19
- B sub-genome	TraesCS3B02G308000.1	3748 (3)	63.95	2210	77.82	1683	86.71
-D sub-genome	TraesCS3D02G273500.1	3718 (3)	65.42	2196	79.33	1683	86.77
*T*. *urartu*	TRIUR3_28465-T1	2088 (3)	75.81	1404	80.84	1404	80.84
*Ae*. *tauschii*	EMT16146	3172 (3)	72.06	1524	85.31	1524	87.53
*O*. *sativa*	OS01T0746400-00	3109 (1)	72	1710	87.66	1710	89.05
*B*. *distachyon*	BRADI2G49670.1	3577 (2)	68	2140	80.13	1713	86.23
*S*. *bicolor*	Sb03g034400.1	3545 (3)	75.91	1867	91.48	1740	94.76
**Dicots**							
*A*. *thaliana*	AT4G32810.1/Max4	3265 (4)	52.8	2026	56.59	1713	61.99
*G*. *max*	GLYMA06G09000.2	3891 (6)	52.72	2049	60.53	1692	65.88
*V*. *vinifera*	VIT_04s0008g03380.t01	2823 (4)	58.83	1782	64.65	1641	67.59
*S*. *lycopersicum*	Solyc08g066650.2.1	3075 (8)	52.36	1907	57.96	1674	61.56
*T*. *cacao*	EOY29749 (TCM_037195)	3680 (9)	52.04	2051	58	1680	63.75
*P*. *trichocarpa*	POPTR_0006s25490.1	3983 (6)	54.84	1674	63.79	1674	64.84
*P*. *persica*	EMJ23585 (PRUPE_ppa006042mg)	2893 (1)	56.11	2436	58.41	1296	67.28
*M*. *truncatula*	AES73861 (MTR_3g109610)	3439 (3)	52.31	2057	57.35	1698	63.28

### Comparative gene structure

**Fig 2:**
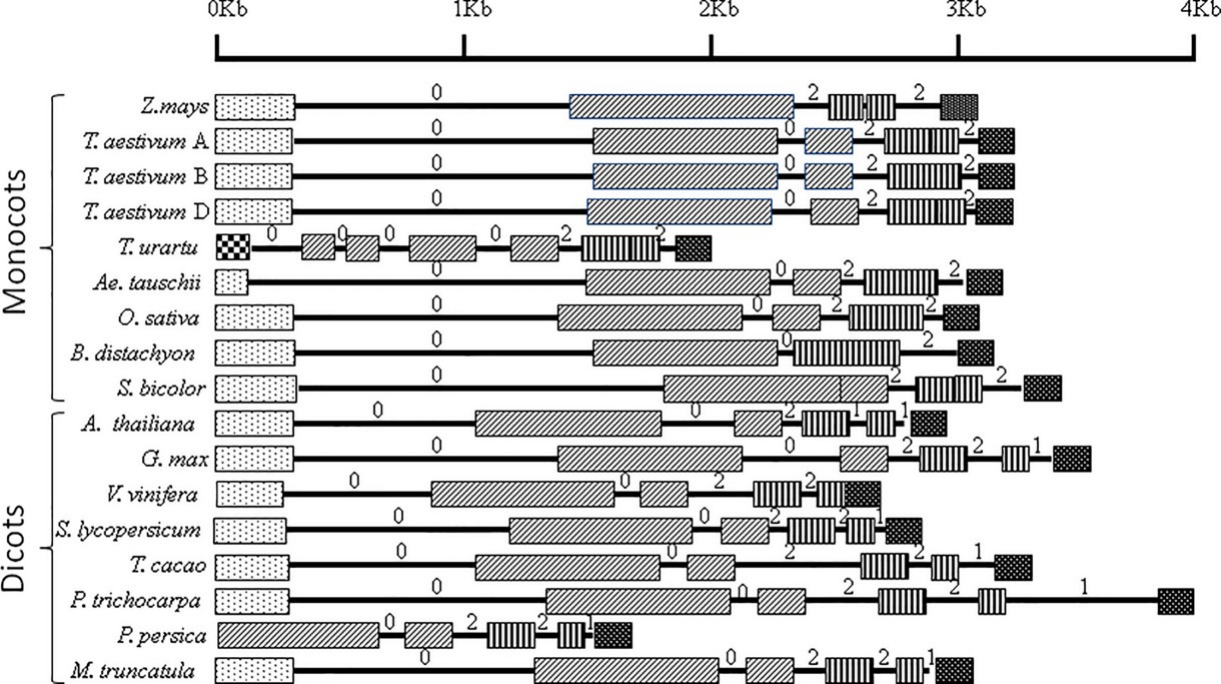
Representation of CCD8 structural genes (from translation start to stop sites) in seven monocots and eight dicots (for wheat, there are three genes, one each on 3A, 3B and 3D chromosomes). Solid patterned boxes indicate exons and lines connecting the exons indicate introns (a box with two patterns represents one exon that resulted due to fusion of two exons from maize *ZmCCD8*). Exons are coded based on the sequence similarity with the respective exons in the *ZmCCD8* (used as reference). Intron phases 0, 1 and 2 are marked above each intron. First exon in *T*. *urartu* (donor of sub-genome A of *T*. *aestivum*) has sequence similarity with a part of 1^st^ intron of *T*. *aestivum*.

The lengths of CCD8 genes ranged from 2.08 kb (*T*. *urartu*) to 3.98 kb (*P*. *trichocarpa*). The differences in lengths were primarily due to variations in number/size of exons/introns and the size of UTRs (Fig 2 and Table 1). The average per cent similarities of cDNA and CDS sequences with those of maize were higher in monocots (cDNA: 77.82–91.48%; CDS: 80.84–94.76%) relative to those in dicots (cDNA: 56.59 to 64.65%; CDS 61.56–67.59%) (Table 1). The observed higher similarity of cDNA and CDS sequences of monocots can be attributed to maize itself being a monocot, which diverged from dicots some 200 MYA [48]. Among the monocots, the per cent similarities of the cDNA and CDS sequences were highest with sorghum because of close evolutionary proximity between sorghum and maize [49]. Among the dicots, the cDNA and CDS sequences of *V*. *vinifera* showed maximum similarity, presumably due to its relatively close relationship with maize.

The number of exons in individual CCD8 genes ranged from 4 to 7, which was also reflected in the number of introns (3/6; Fig 2 and S1 Table) and may be attributed to gain/loss of introns. Similar results for genes encoding AGPase enzymes were earlier reported by us [29]. Of particular interest are the CCD8 genes of *T*. *urartu*, *Ae*. *tauschii* and *P*. *persica*, where the structure of CCD8 genes deviated significantly from the general pattern. The CCD8 gene of *T*. *urartu* (progenitor of the A genome of wheat) surprisingly deviated from that of the CCD8 gene from A sub-genome of bread wheat; the first 396 base pairs (encoding 132 amino acids) differed in CCD8 genes of *T*. *urartu* and *T*. *aestivum* sub-genome A. The CCD8 of *T*. *urartu* also carried a deletion of 240 bp, and the first exon of *T*. *urartu* (156 bp) resembled a part of first intron of CCD8 of *T*. *aestivum*. Apparently, these deviations in CCD8 structure of *T*. *urartu* occurred after the incorporation of A genome of *T*. *urartu* in 4x/6x wheat. Further, the deviations in *T*. *urartu* also included deletions of segments of coding sequences leading to deletions of amino acids at postions 192–194, 201–202, 207–212, 217–219 and 275–304. A deletion of 180 bp encoding 60 aa (72–131 aa) also occurred in CCD8 gene of *Ae*. *tauschii*, the donor of the D genome. In CCD8 gene of *P*. *persica* also, the first exon, first intron and a part of the second exon were absent.

Relative to the reference CCD8 gene of *Z*. *mays*, both loss and gain of introns was observed in CCD8 genes of all the other species, except *S*. *bicolor*, where the intron and exon numbers were similar to those of the *Z*. *mays*. Similar gain and loss of introns was also reported earlier in some other cases [50–51]. The intron/exon boundaries (splice sites) did not differ from the known conserved GT/AG boundaries [52]. Intron phases included all the three phases, the intron phase 1 was absent in monocots and relatively infrequent in dicots (phase 1 being 20% as against phase 0 being 40%). These results suggest that only 40% intron phases (phase 0 = 40%) are conserved, which allowed conservation of codons in the reading frame [53]. However, *A*. *thaliana*, *V*. *vinifera* and *P*. *persica* did not follow this general pattern (Fig 2). The intron insertion sites and the intron phases in monocots did not differ from those in the ancestral genome [54].

Individual exons and introns differed in size, the length of exons being 103–938 bp and that of introns being 41–1471 bp. The total length of exome ranged from 1296 bp to 1740 bp (Fig 2 and S1 Table). The average sequence similarity for exons was higher in monocots (81.67 to 94.79%) than in dicots (60.86 to 66.53%) (S2 Table).

The average Ka/Ks value for CCD8 genes was 3.3 (S3 and S4 Tables), suggesting that CCD8 genes have undergone positive selection during speciation [55].

### Synteny/Collinearity analysis

Analysis of synteny conservation was undertaken using a block of 31 genes, including 15 genes flanking either side of the CCD8 gene on *Z*. *mays* chromosome 3 (S5 Table), which corresponds to wheat homoeologous group 3, rice chromosome 1, and Brachypodium chromosome 2 [56–57]. This analysis was possible for only 13 of the 15 species, since the genome sequences of *T*. *aestivum* and *M*. *truncatula* could not be utilized by Genomicus. Some degree of synteny conservation was observed among *Z*. *mays*, *S*. *bicolor*, *B*. *distachyon* and *O*. *sativa* (S1 Fig). Out of the 30 genes flanking the *ZmCCD8*, only 12 genes were syntenous in *S*. *bicolor* and *B*. *distachyon* each and 8 genes were syntenous in *O*. *sativa*. Even in these three species (*S*. *bicolor*, *B*. *distachyon* and *O*. *sativa*), the collinearity within the synteny block was rather disrupted. Some degree of synteny observed in the present study is in agreement with an earlier study, where CCD8 genes of these species were reported to occupy orthologous positions [6]. The loss of shared synteny in most of the species may be attributed to rearrangements in the genomes during the course of evolution [58]. This partial syntenic relationship was further confirmed through a study of the level of orthology between maize, wheat, rice and Brachypodium.

### SSRs and retro-elements

As many as 24 SSRs were detected in different regions (exons, introns, UTRs) of CCD8 genes of 12 of the 15 species examined (no SSRs were available in CCD8 genes of *O*. *sativa*, *V*. *vinifera* and *S*. *lycopersicum*). The repeat units in SSRs ranged in size from 1–7 (mononucleotide repeats to heptanucleotide repeats), and the number of SSRs per CCD8 gene ranged from 1 to 4 (for details, see S6 Table). As known, the presence of SSRs within genes can lead to (*i*) a gain or loss of gene function, (*ii*) affect transcription and translation, (*iii*) mRNA splicing, or (*iv*) export to cytoplasm. All these effects eventually lead to phenotypic changes [59].

Retro-elements in the form of a solitary SINE element and a solitary Helitron occurred in only one species, namely *P*. *trichocarpa*; no other species carried any retro-elements. The polymorphic SSRs and the polymorphic retro-elements may be utilized for developing markers for use in marker-aided breeding programmes aimed at improvement of either shoot branching or phosphate use efficiency in crops like wheat.

### Promoter analysis

**Fig 3:**
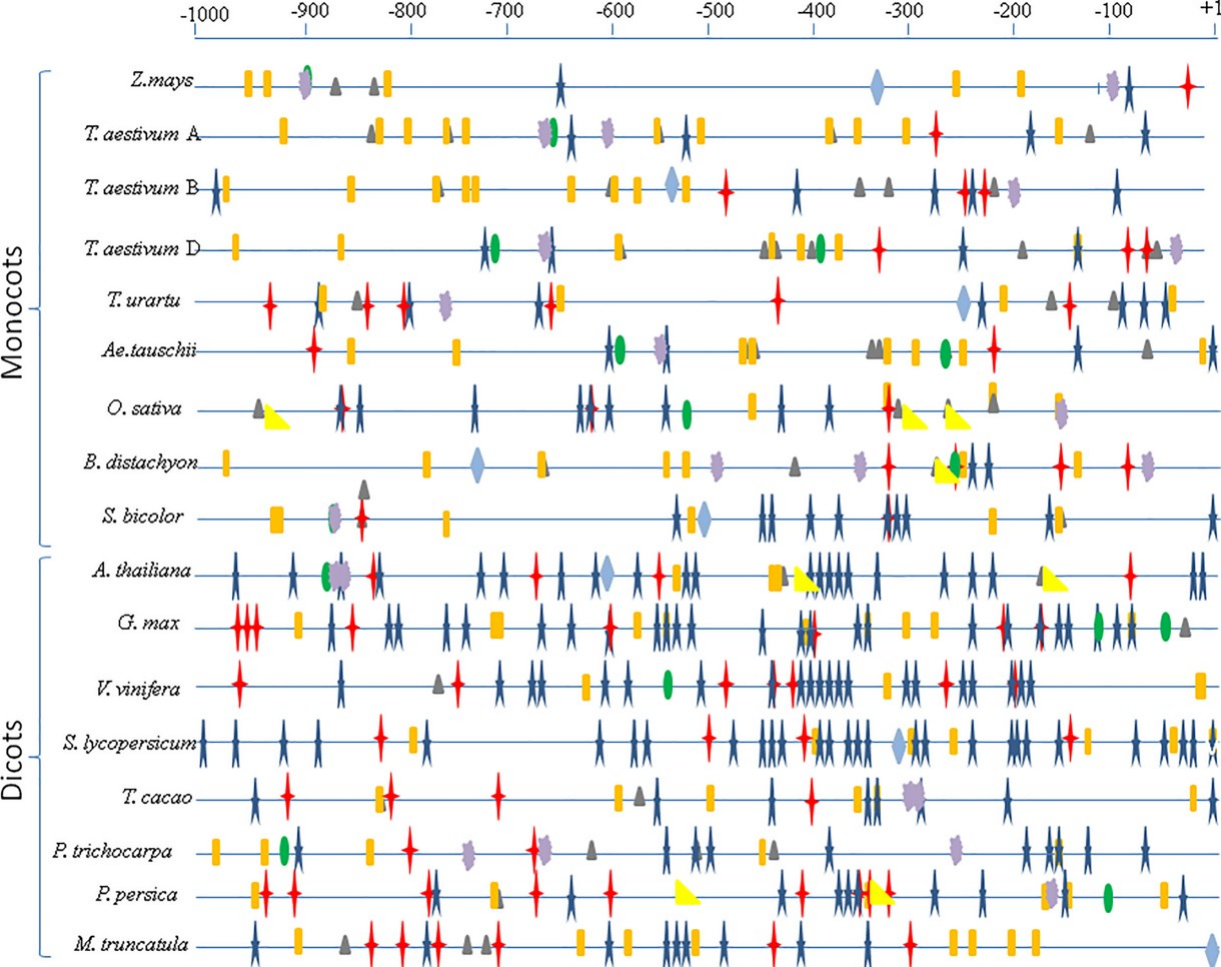
Regulatory elements identified in 1 kb upstream region of the translation start site (ATG) of CCD8 genes. Different symbols indicate major regulatory elements. TATA box (star), CAAT box (plus), light response elements (rectangle), abiotic stress response elements (triangle), biotic stress response elements (dispersed), endosperm-specific response elements (oval), auxin response elements (diamond) and gibberellic acid response elements (right angle triangle).

Promoter analysis allowed identification of some cis-acting regulatory elements (TATA, CAAT) that were common to all genes. Other cis-acting elements included light response elements (Sp1, Box 4, Box 2, MNF1, G-Box, GAG motif, GT1, TCT, I-Box, ATCT motif, 3-AF1, ACE) and response elements for tissue-specificity (Skn_1 and GCN4); multiple copies of some response elements were also common in the promoter regions of some CCD8 genes (Fig 3). These cis-elements should impart light responsive and tissue specific expression of CCD8 genes during plant development [60].

The presence of response elements for auxin (TGA, Aux-RR) and gibberellic acid (GARE, P-Box, TAGTTA) in the promoter regions of CCD8 genes in some of the species suggested that the expression of CCD8 genes and SL level is also regulated by the presence of other hormones. Recent studies demonstrated that auxin and strigolactone modulate the levels and distributions of each other, thus forming a dynamic feedback loop between the two hormones [61]. There were four response elements for biotic (CGTCA motif, TCA, TGACG element, MBSII) and the same number for abiotic stresses (ABRE, MBS, TC-rich repeats, CCAAT, HSE). This confirmed that the level of SLs is regulated by a variety of stresses including low P conditions, drought, salinity and plant-microbe interactions [62].

### Protein sequence and structural analyses

#### Primary structure

The length of predicted CCD8 proteins ranged from 431 amino acids (aa) in *P*. *persica* to 579 aa in *S*. *bicolor* (Table 2). This range matches exactly the range in the size of exomes (1296–1740 bp; 3 bp = one amino acid) recorded in the gene structure described above in this paper (Fig 2 and S1 Table). The difference of a triplet between the coding sequence and a single aa in the translated product is primarily due to the termination codon, which is a part of exome but does not code for any aa.

**Table 2:** Details of CCD8 proteins and their conserved domains in 15 selected species.

Species	Ensembl Plants Id's	Protein		Protein domain RPE65	
		Length in aa	Similarity (%)	Position; length in bp	Similarity (%)
**Monocots**					
*Z*. *mays*	Zm00001d043442_T001	572	100	103–564; 462	100
*T*. *aestivum* -A sub-genome	TraesCS3A02G274300	560	84.35	93–555; 463	84.27
-B sub-genome	TraesCS3B02G308000.1	560	84.71	93–555; 463	84.81
-D sub-genome	TraesCS3D02G273500.1	560	84.35	93–555; 463	84.45
*T*. *urartu*	TRIUR3_28465-T1	467	76.87	51–462; 412	82.34
*Ae*. *tauschii*	EMT16146	507	86.19	49–502; 454	86.17
*O*. *sativa*	OS01T0746400-00	569	86.99	99–561; 463	86.07
*B*. *distachyon*	BRADI2G49670.1	570	84.11	104–565; 462	84.11
*S*. *bicolor*	Sb03g034400.1	579	93.71	110–571; 462	94.75
**Dicots**					
*A*. *thaliana*	AT4G32810.1/Max4	570	60.87	92–566; 475	61.24
*G*. *max*	GLYMA06G09000.2	563	69.08	97–559; 463	69.1
*V*. *vinifera*	VIT_04s0008g03380.t01	546	70.66	80–542; 463	72.08
*S*. *lycopersicum*	Solyc08g066650.2.1	557	69.02	90–553; 464	70.43
*T*. *cacao*	EOY29749 (TCM_037195)	559	66.85	92–555; 464	67.96
*P*. *trichocarpa*	POPTR_0006s25490.1	557	71.25	91–553; 463	71.72
*P*. *persica*	EMJ23585 (PRUPE_ppa006042mg)	431	76.1	1–427; 427	76.1
*M*. *truncatula*	AES73861 (MTR_3g109610)	565	68.17	98–561; 464	68.91

aa, amino acid

**Fig 4:**
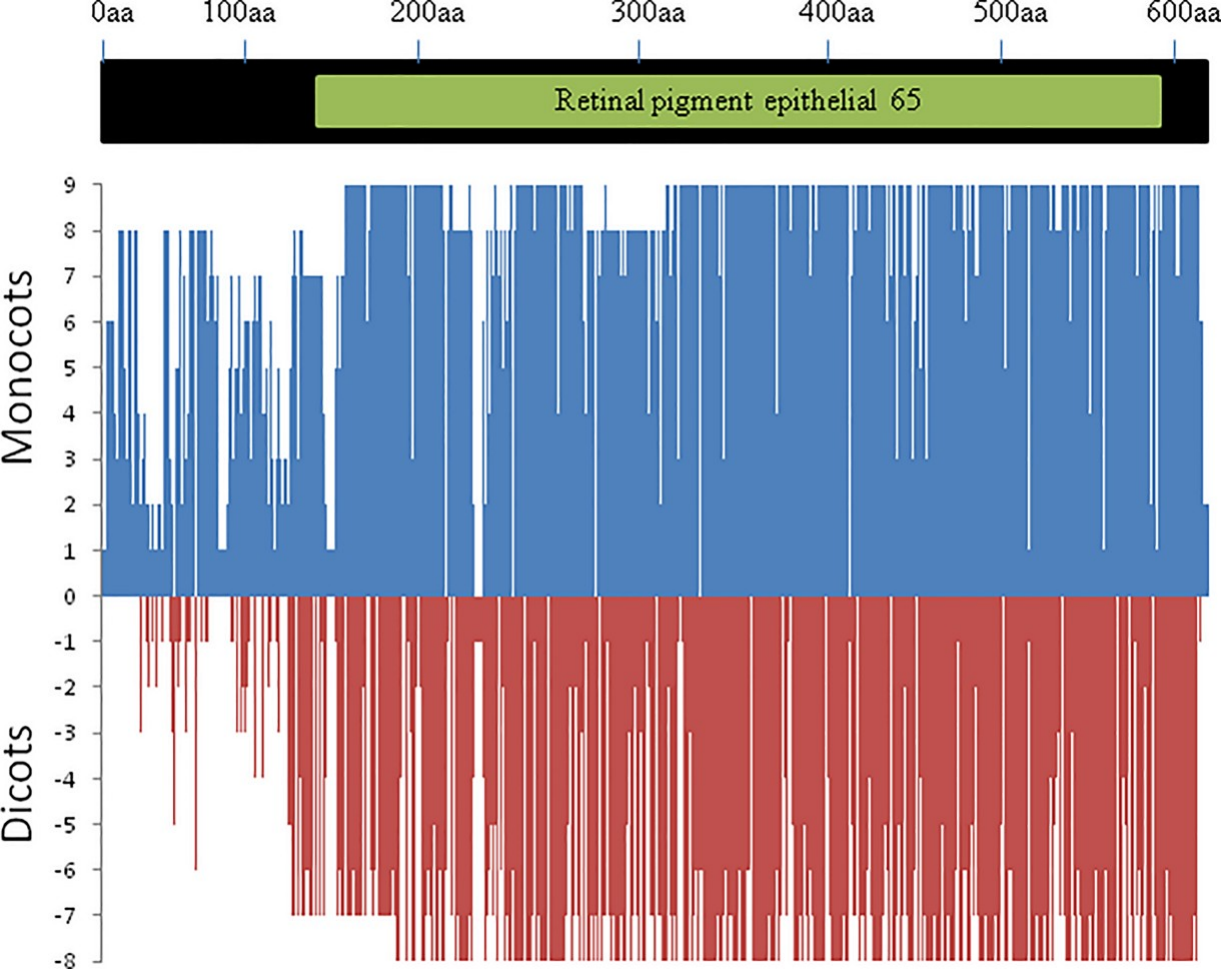
Amino acid sequence similarity of CCD8 proteins among seven monocots and eight dicots with respect to consensus sequence (for wheat, there are three CCD8 proteins derived one each from 3A, 3B and 3D chromosomes). Position 0 (on y-axis) indicates amino acid (aa) consensus sequence. Presence of similar aa residues against consensus is plotted on a scale of 1 to 9 in monocots and -1 to -8 in dicots. Consensus protein on top is indicated by solid-bar; a small-bar within solid-bar indicates the position of the conserved domain.

The variations in the length of CCD8 proteins were mainly due to deletions, insertions and mismatches, particularly in the hypervariable N-terminal region (up to ~70 aa), which corresponds to the variation in gene structure described above in this paper (comparative gene structure section). Another conspicuous deviation was observed in CCD8 protein of *P*. *persica*, where absence of ~125 aa residues also corresponds to the deviation in CCD8 gene of this species. Maximum number of extra aa residues were present in *A*. *thaliana* CCD8 protein. For instance, valine, isoleucine, asparagine, histidine, tryptophan, aspartic acid were present at positions 203–206 (isoleucine at positions 204 and 205), 274, 325 and 406, respectively (Fig 4 and S2 Fig).

A single conserved domain that occurred in the consensus sequence (116–592 aa) belongs to RPE65 super-family (RPE65 = retinal pigment epithelium-specific 65), which was also a characteristic feature of CCD8 proteins of all the species examined. Among all these species, the size of this domain was conserved ranging from 412 aa to 475 aa (Table 2). The similarity of this domain with the maize CCD8 domain was higher in monocots (82.34 to 94.75%) than in dicots (61.24 to 76.10%) (Table 2). RPE65 domain is a characteristic feature of all the enzymes (including CCD8) that are involved in the biosynthesis of apocarotenoids, which are the intermediate products in the synthesis of strigolactones catalysed by CCD8. Therefore, the role of the domain RPE65 in CCD8 is rather general in nature, and not specific; this is known to be a common feature in many enzymes that are involved in a variety of biosynthetic pathways.

#### Physico-chemical analysis

The molecular weight of CCD8 proteins ranged from 47.66 to 63.96 kD. The total number of negatively charged aa residues exceeded the total number of positively charged aa residues. The isoelectric point was within the acidic range (5.75–6.99) indicating that the proteins encoded by CCD8 genes of all the selected species are sparingly soluble in aqueous medium [63]. The aliphatic index (relative volume occupied by aliphatic/hydrophobic aa: glycine, alanine, valine, isoleucine, and leucine) was relatively higher in dicots (78.06–83.29) than in monocots (75.13–78.73). It has been reported that higher aliphatic index is correlated with stability of proteins at wide range of temperatures [64]. Based on values of aliphatic index, the results of the present study suggested relatively higher stability of the proteins encoded by the dicots. The value for Grand Average Hydropathy (GRAVY, which represents the hydrobhobic and hydrophilic nature of aa) was higher in monocot proteins (range: -0.252 to -0.349) relative to dicot proteins (range: -0.313 to -0.366 except *P*. *persica*) (S7 Table). The CCD8 proteins of all monocots were found to be unstable except those associated with the B sub-genome of *T*. *aestivum* and *Ae*. *tauschii*. In dicots, the CCD8 proteins were stable except in *G*. *max*. Overall, the results indicated that the proteins encoded by the monocots had a relatively low level of stability, which might make it difficult to obtain these proteins in pure crystalline state for a study of their crystalline structure [65].

#### Secondary structure and motif search

A comparison of the secondary structures of CCD8 proteins in 15 species suggested that random coils dominated in the secondary structure followed by helices except for *Z*. *mays* and *T*. *urartu* among monocots and *G*. *max*, *T*. *cacao*, *P*. *persica* and *M*. *truncatula* among dicots (S8 Table and S3 Fig). The random coils are known to form irregular structured regions permitting polypeptide chain to fold in a unique way. These results support that the proteins encoded by CCD8 genes tend to attain a globular structure which is known to be highly stable structure.

As many as 12 motifs were identified using MEME; these motifs ranged from 15 aa to 50 aa in most species except *T*. *urartu* (motifs 10,11 and 12 absent), *Ae*. *tauschii* (motif 12 absent), *A*. *thaliana* (motif 11 absent) and *P*. *persica* (motifs 9 and 12 absent) (for details, see S9 Table). Only six of these 12 motifs (motifs 1, 2, 4, 5, 9 and 10 related to carotenoid oxygenase family) were earlier characterized using Interpro Scan search (IPR004294); all other motifs were novel, which may be validated in future studies to identify their role in providing active sites for binding of regulatory proteins.

#### 3D structures

The percent identity of 3D structures of the CCD8 proteins with the chosen template (PDB id: 5kk0.1.A) was low (23.90–32.80), which may be attributed to the non-availability of X-ray crystallographic structure of CCD8 protein in case of plants. The 3D structures had a high level of confidence, with Global Model Quality Estimation GMQE) score ranging within the acceptable range of 0.40 to 0.60; the higher value indicated higher reliability [66] (S10 Table).

The use of Ramachandran plot for evaluation of the accuracy of protein structures is widely known and has been emphasized in several recent studies [67]. In the present study, most aa residues were present in the favoured region of Ramachandran plots relative to those falling in the ‘allowed’ and ‘disallowed’ regions. Several features including the high values of quality factors through ERRAT and 3D-1D score by VERIFY3D and the values of *Dfire energy* (lower values indicate better quality structures) indicated that the modelled structures were of good quality. These can, therefore, be used for further analysis (S10 Table).

### Simulation analysis of 3D models

Rg values ranged from 1.6 nm to 2.4 nm, except for the protein model belonging to *B*. *distachyon*, which had Rg value >4.1nm, suggesting that protein models were generally stable. When a two dimensional plot was drawn taking into consideration Rg values with respect to the simulation time, no changes were noticed in the Rg values, suggesting their stability over time also (S4 Fig). Overall, the data suggested stable folded state during 10 nsec simulation time. Based on root mean square deviation (RMSD) data also, protein models were found to be constant and stable (range: 0.3–1.3Å). Only in *T*. *urartu* and *Ae*. *tauschii*, the protein models showed higher RMSD values, although these values were still in the acceptable range (0.5–2.0Å) (S5 Fig).

### Superimposition and alignment of 3D structures

**Fig 5:**
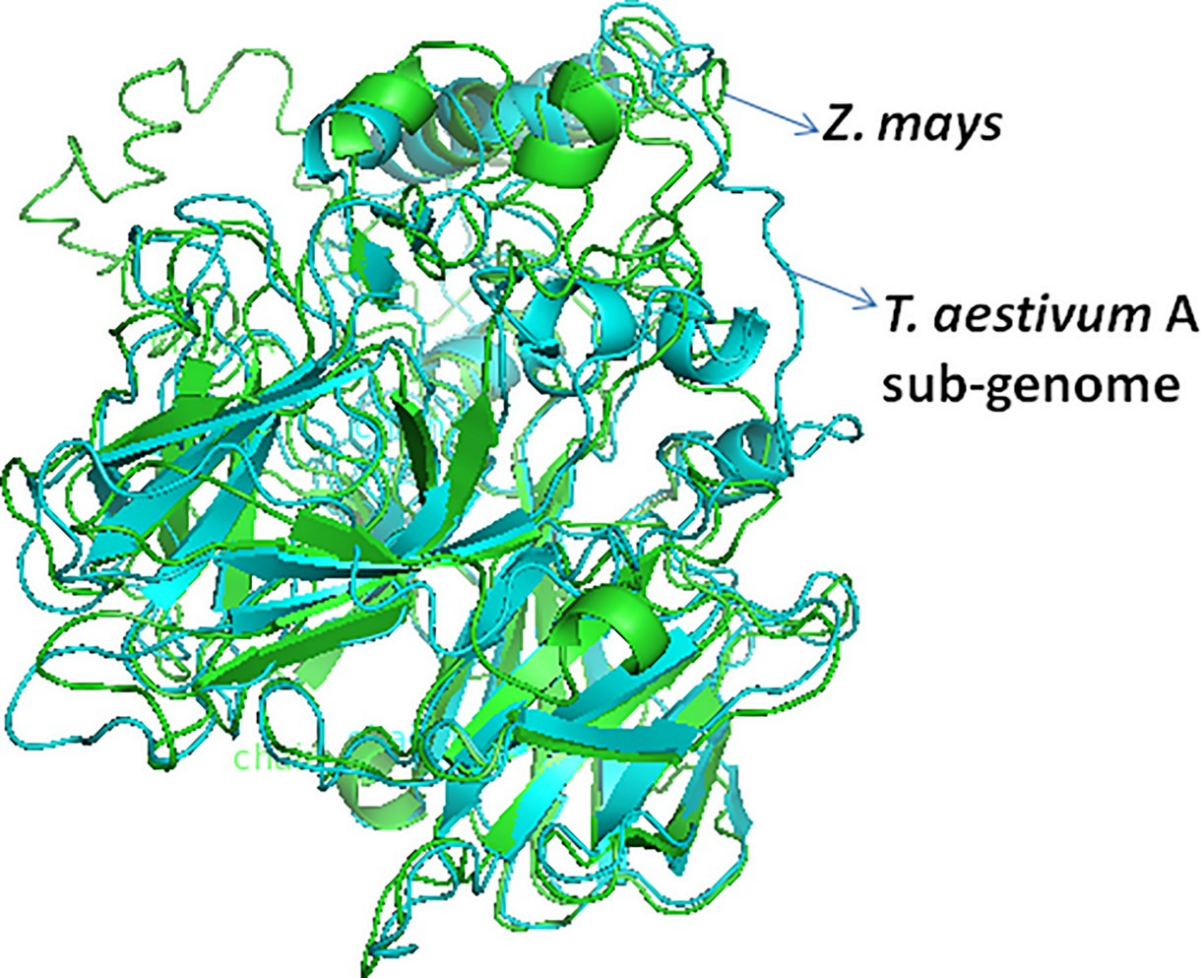
Representative figure showing superimposed structure of the predicted 3D structure of *T*. *aestivum* CCD8 protein belonging to A sub-genome over 3D structure of *Z*. *mays* CCD8 protein.

The predicted values of different parameters were obtained through superimposition of the 3D protein structures (with minimized energy) for each species over the corresponding 3D protein structure of maize (Fig 5 and S11 Table). The aligned protein structures showed a fairly high level of similarity, which ranged from 50.98 to 85.19%. Physico-chemical properties also showed high level of similarity ranging from 44.12 to 78.43%. The values for RMSD were 1.72 to 3.26 Å, suggesting that the average distance of all pairs of residues in two structures were high, perhaps because a local error can arise in a big RMSD value, although the global topology is correct [68].

### Sub-cellular localization, functions and ligand binding sites

CCD8 proteins of all species were largely located in the plastids, as also shown in previous studies in maize, sorghum, rice and Arabidopsis [1,22]. It is also reported that the CCDs are involved in the remodelling of carotenoids and therefore often targeted to plastids [69].

Functional annotation (using gene ontology analysis) suggested that CCD8 proteins are involved in oxidation-reduction process. They have their characteristic role in oxidoreductase activity, dioxygenase activity and in metal-ion binding [1,69] (S12 Table).

**Fig 6:**
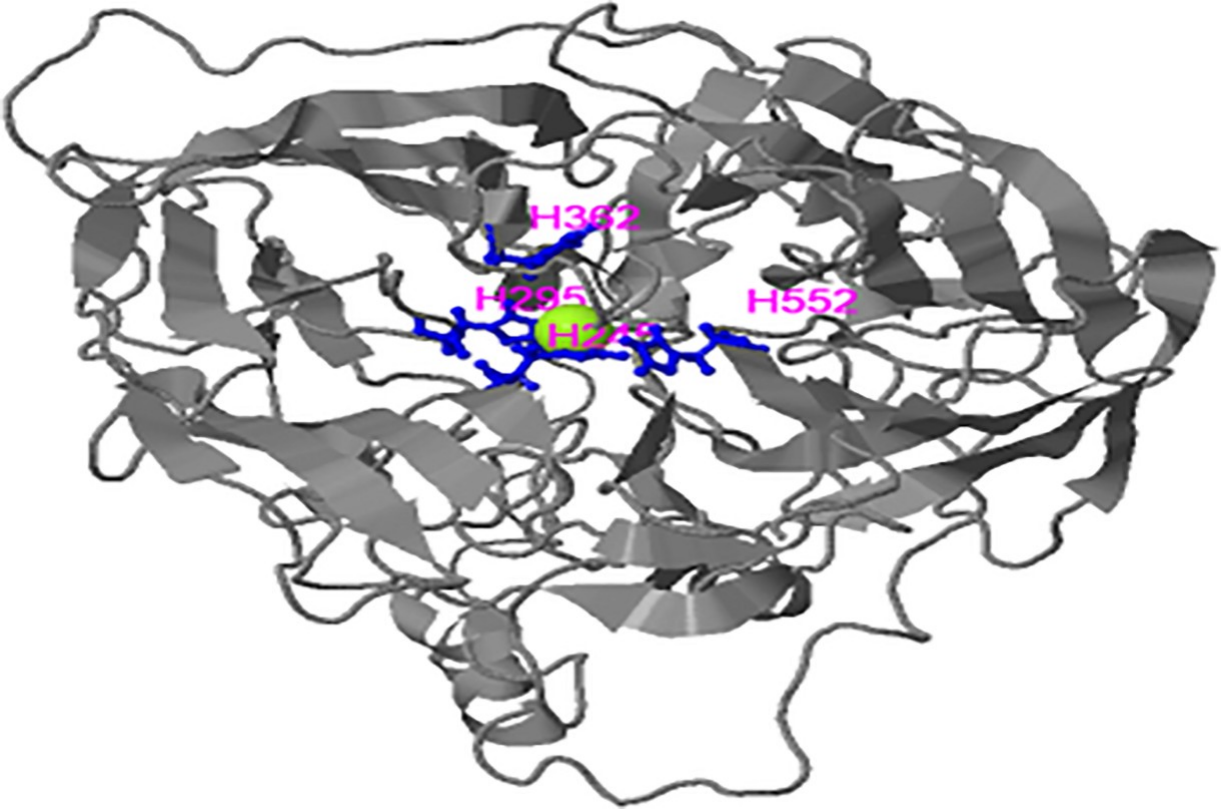
3D structure of CCD8 protein of *T*. *aestivum* belonging to A sub-genome. The four conserved histidine residues and their positions are shown in middle. Solid sphere represents iron catalytic center.

CCD8 proteins are already known to have Fe^++^ binding sites. During the present study, 10–14 clusters of ligands were predicted to bind with the Fe^++^ binding catalytic centre in CCD8 proteins. The binding sites were predicted to have four conserved histidine residues. This is a characteristic feature of apocarotenoid oxygenase enzyme (ACO) of *Synechocystis* [69] and found in majority of species examined (Fig 6). The conserved His residues/ligand binding sites were confined to the characteristic domain of CCD8 proteins. However, in *T*. *urartu*, 4^th^ conserved histidine residue, and in *M*. *truncatula*, 2^nd^ conserved histidine residue were absent, thus leaving only three histidine residues at the binding sites.

### Phylogenetic analysis

**Fig 7:**
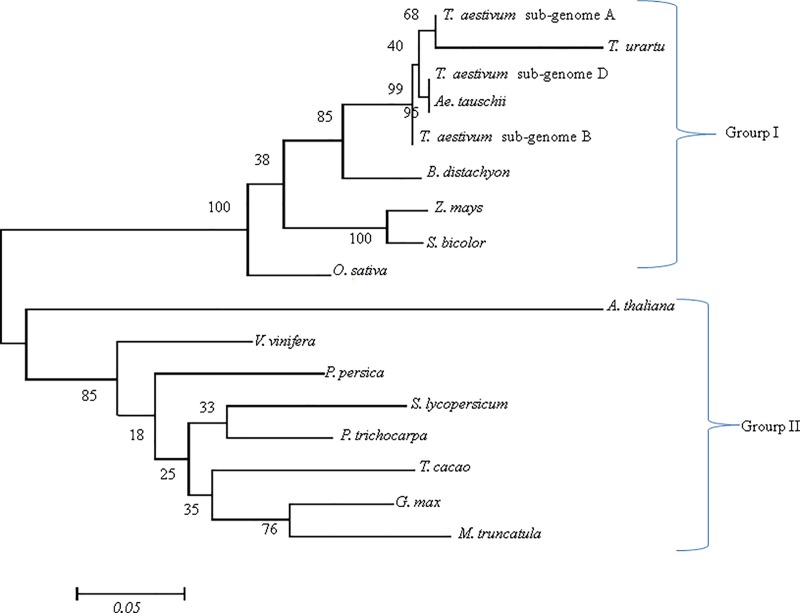
Phylogenetic tree obtained by maximum-likelihood method using amino acid sequences of CCD8 proteins of seven monocots and eight dicots depicting the relationship among monocots and dicots (for wheat there are 3 proteins for three homoeologues). The branch length represents magnitude of genetic change.

The phylogenetic tree (based on aa sequences) had two major groups, Group I with all the monocots, and Group II with all the dicots. Among the monocots, as expected the sub-genome A of *T*. *aestivum* was close to *T*. *urartu* and *T*. *aestivum* sub-genome D was close to *Ae*. *tauschii*, whereas *Z*. *mays* was close to *S*. *bicolor* and among dicots, *S*. *lycopersicum* was close to *P*. *trichocarpa*, and *G*. *max* was close to *M*. *truncatula* (Fig 7). Phylogenetic analysis supports the conclusion that CCD8 genes in monocots and dicots diverged early in the evolutionary history. This was also inferred in earlier studies where monocots and dicots also made two separate clusters [23,24].

### *In silico* expression analysis

The *in silico* expression analysis on the basis of microarray data was available for only seven species (*Z*. *mays*, *T*. *aestivum*, *O*. *sativa*, *B*. *distachyon*, *A*. *thaliana*, *G*. *max* and *M*. *truncatula*), which suggested that the level of expression was generally high in the vegetative tissues relative to reproductive tissues (S6 Fig). Abundance of CCD8 in terms of SLs in all the vegetative tissues and at all the developmental stages (S7 Fig) may be attributed to the transport of SLs to aerial parts of the plants after their synthesis in roots. These are in turn involved in important functions like leaf senescence, shoot branching/tillering etc., which suggests tissue and development stage specific expression of CCD8. Future studies may provide further clues to the role of SLs in the development during reproductive phase.

Under phosphate limited conditions, the expression was upregulated in *M*. *truncatula* only (fold change: >2 and P value-0.5), whereas, in *T*. *aestivum* and *O*. *sativa*, no differential expression was found in controlled vs. experimental conditions (S8 Fig). The data on P stress was not available for rest of the species. No expression data in reponse to biotic or abiotic stresses was available except in maize where synthesis of CCD8/SLs was found to be upregulated due to drought stress [6]. Similar results were also reported in *A*. *thaliana* where the role of SLs in positive regulation was examined under drought and salt stress using mutant and microarray analysis [70]. Therefore, a future study of the role of available regulatory sequences in response to biotic and abiotic stresses may prove rewarding, as suggested by some preliminary results obtained in the present study (see next).

### qRT-PCR expression analysis of TaCCD8 genes

**Fig 8:**
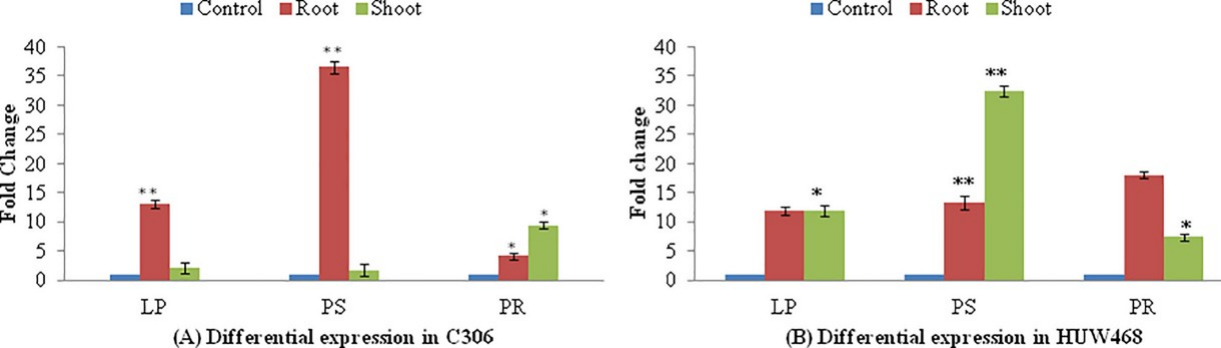
Combined relative expression level of three TaCCD8 genes (located on chromosomes 3A, 3B and 3D) in root and shoot tissues of wheat seedlings of cv. C306 (A) and cv. HUW 468 (B). Transcript levels were normalized with respect to expression of *TaAct* mRNA. Each data point represents mean ± SE (n = 3). Significance of combined expression level of three TaCCD8 genes (located on chromosomes 3A, 3B and 3D) in different treatments vs. the control was tested using *t-*test; *, ** represents significance at p < 0.05 and p < 0.01, respectively. C, control; LP, low P; PS, P starvation and PR, P restoration.

Two wheat cvs. (C306 and HUW468) were used for wet-lab experiments: of these two wheat cvs., C306 is known to be relatively tolerant to abiotic stresses like drought, and has a low nitrogen use efficiency (NUE). Significant differences in expression patterns of TaCCD8 genes were observed under three different P regimes (Fig 8). Following results were observed: (i) In root tissue of cv. C306, ~13 fold increase in expression of TaCCD8 genes was observed under low P, whereas ~37 fold increase in expression was observed under P starved condition. In root tissue of cv. HUW468, the expression of the gene was >10 fold higher at all the three P regimes but the maximum expression of the gene was observed after P restoration. (ii) In shoot tissue, maximum expression of TaCCD8 in cv. C306 was observed after P restoration (~9.5 fold), and that in cv. HUW468 increased ~12 fold under low P and ~33 fold under P starvation.

The expression pattern of TaCCD8 genes in root and shoot tissues seems to be in agreement with the results reported in previous studies [6]. The expression of CCD8 gene in Arabidopsis, pea, petunia, tomato, tobacco and potato was reported to be predominant in root tissue; in rice and chrysanthemum, the expression of this gene was reported to be high in stem tissue [21]. Since the combined expression of TaCCD8 genes was higher in roots of cv. C306 and shoots of cv. HUW468 under P starved conditios, it may be concluded that the expression of these genes is not only tissue specific, but also genotype-dependent [21,71]. Increased expression of TaCCD8 genes was observed under limited supply of phosphate. This is consistent with the role of CCD8 genes in nutrient uptake through SLs biosynthesis. Keeping this in view, future studies on expression using wheat mutants for CCD8 genes in root and shoot tissues under limited supply of phosphate may prove rewarding.

## Supporting information

S1 Fig Representative figure showing synteny and collinearity of a block of 31 genes of *Zea mays* (15 genes on either side of *ZmCCD8* gene) with respective genes of *S*. *bicolor*, *B*. *distachyon* and *O*. *sativa*. The numbers given above the genes correspond to the gene number given in S5 Table.
(TIF)

S2 Fig Multiple sequence alignment of CCD8 proteins of all the selected 15 species and their comparison with the CCD8 consensus protein sequence. The figure shows insertions, deletions and mismatches in the CCD8 protein of the individual species with respect to the consensus sequence.
(JPG)

S3 Fig Representative figure showing secondary structure of protein sequence encoded by CCD8 gene of *Z*. *mays*. (TIF)

S4 Fig Radius of gyration plot for each of the 17 simulated CCD8 proteins belonging to seven monocots and eight dicots (for wheat there are 3 proteins for three homoeologues). (TIF)

S5 Fig RMSD plot of CCD8 protein models of seven monocots and eight dicots representing protein backbone atoms using 1 to 10 nsec trajectory data (for wheat there are 3 proteins for three homoeologues). (TIF)

S6 Fig Representative figure showing *in silico* expression of TaCCD8 in different tissues of wheat. (TIF)

S7 Fig Representative figure showing *in silico* expression of TaCCD8 at different development stages of wheat plant. (1) germination, (2) seedling growth, (3) tillering, (4) stem elongation, (5) booting, (6) inflorescence emergence, (7) anthesis, (8) milk stage, (9) dough development stage, and (10) ripening.
(TIF)

S8 Fig *In silico* expression of CCD8 genes belonging to *Oryza sativa*, *Triticum aestivum* and *Medicago truncatula* at different dose of phosphorous. (TIF)

S1 Table **Details of the positions of exons (upper row) and introns (lower row) of CCD8 structural gene sequences (in bp) in 15 different species.** The position of first exon is marked from translation start site.
(DOCX)

S2 Table Per cent similarity of exons in CCD8 genes of 14 different species with respect to *Z*. *mays*. (DOCX)

S3 Table **Values of non-synonymous substitutions (Ka; upper row) and synonymous substitutions (Ks; lower row) in CCD8 genes of seven monocots (including homoeologues on group 3 chromosomes of wheat)**.
(DOCX)

S4 Table **Values of non-synonymous substitutions (Ka; upper row) and synonymous substitutions (Ks; lower row) in CCD8 genes of eight dicots**.
(DOCX)

S5 Table Details of 31 genes (including the CCD8 gene of *Z*. *mays* at position 16) used for synteny and collinearity analysis. (DOCX)

S6 Table Simple sequence repeats (SSRs) and retro-elements identified in CCD8 genes belonging to 12 species. (DOCX)

S7 Table Primary protein sequence analyses of CCD8 proteins of 15 selected species. (DOCX)

S8 Table Analysis of secondary structure of CCD8 proteins of selected 15 species. (DOCX)

S9 Table Predicted values of different parameters for the motifs identified in CCD8 proteins using MEME suite. (DOCX)

S10 Table Details of 3D structures of CCD8 proteins (using Swiss-model) and their quality assessment parameters in selected 15 species obtained using SAVES and structure assessment tool of Swiss-Model. (DOCX)

S11 Table Predicted values of different parameters obtained after superimposition of 3D protein structures of CCD8 of selected 14 species over 3D protein structure of CCD8 of maize. (DOCX)

S12 Table Predicted scores of different parameters related to cellular component, biological processes and biochemical functions of CCD8 proteins of all the selected 15 species obtained through their functional analysis. (DOCX)
