# Supplementary figures and images for: A study of CCD8 genes/proteins in seven monocots and eight dicots
Source: PLoS One. 2019 Mar 12;14(3):e0213531. doi: 10.1371/journal.pone.0213531 (PMC6413960; doi:10.1371/journal.pone.0213531)

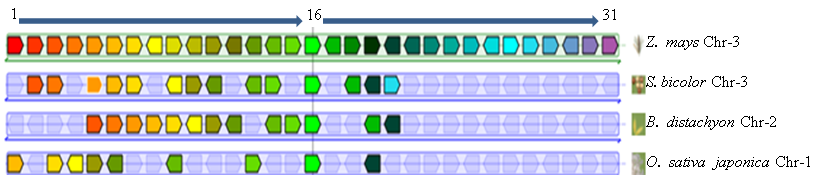

Supplement: S1 Fig — The numbers given above the genes correspond to the gene number given in S5 Table. (TIF) [file pone.0213531.s001.tif]

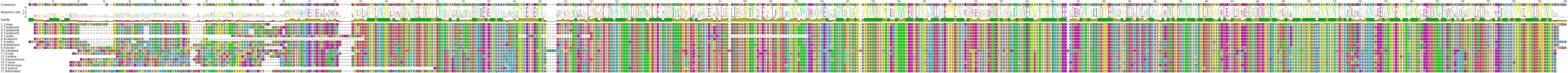

Supplement: S2 Fig — The figure shows insertions, deletions and mismatches in the CCD8 protein of the individual species with respect to the consensus sequence. (JPG) [file pone.0213531.s002.jpg]

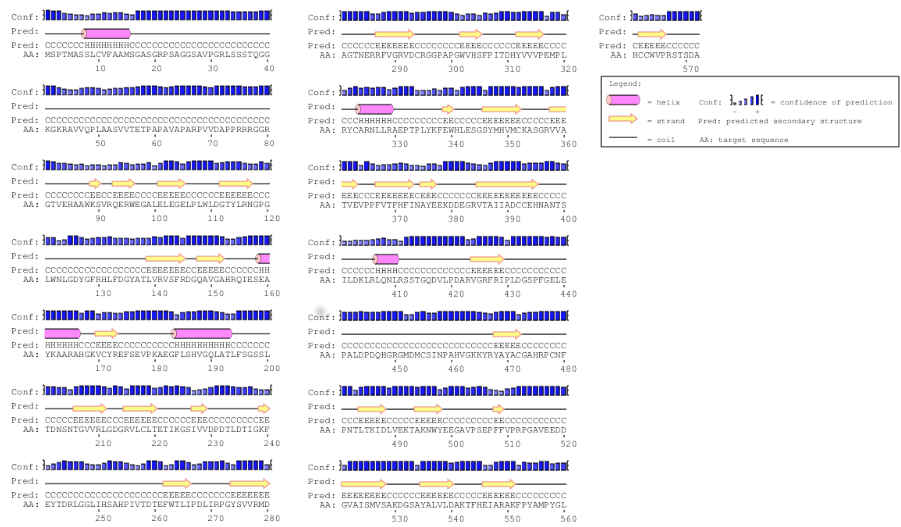

Supplement: S3 Fig — (TIF) [file pone.0213531.s003.tif]

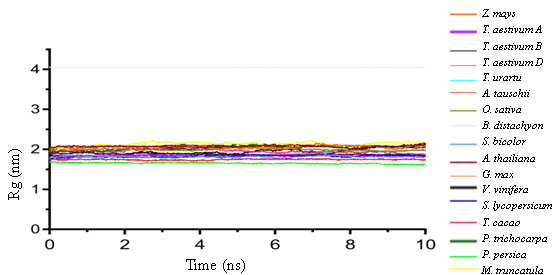

Supplement: S4 Fig — (TIF) [file pone.0213531.s004.tif]

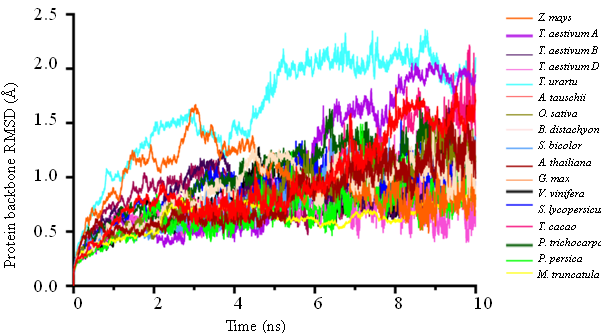

Supplement: S5 Fig — (TIF) [file pone.0213531.s005.tif]

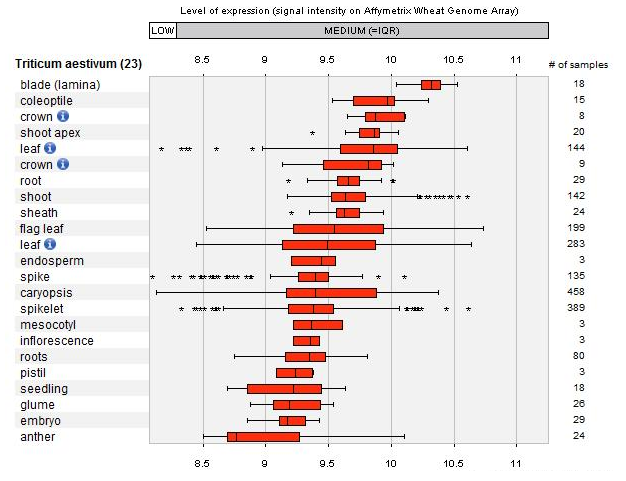

Supplement: S6 Fig — (TIF) [file pone.0213531.s006.tif]

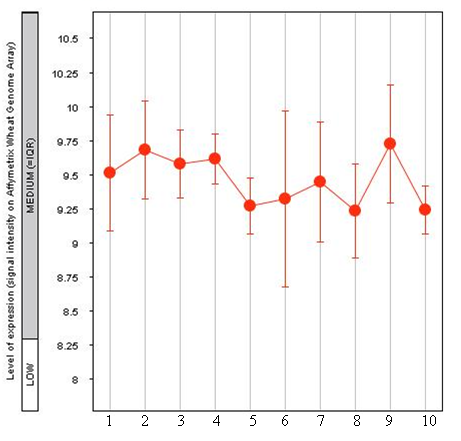

Supplement: S7 Fig — (1) germination, (2) seedling growth, (3) tillering, (4) stem elongation, (5) booting, (6) inflorescence emergence, (7) anthesis, (8) milk stage, (9) dough development stage, and (10) ripening. (TIF) [file pone.0213531.s007.tif]

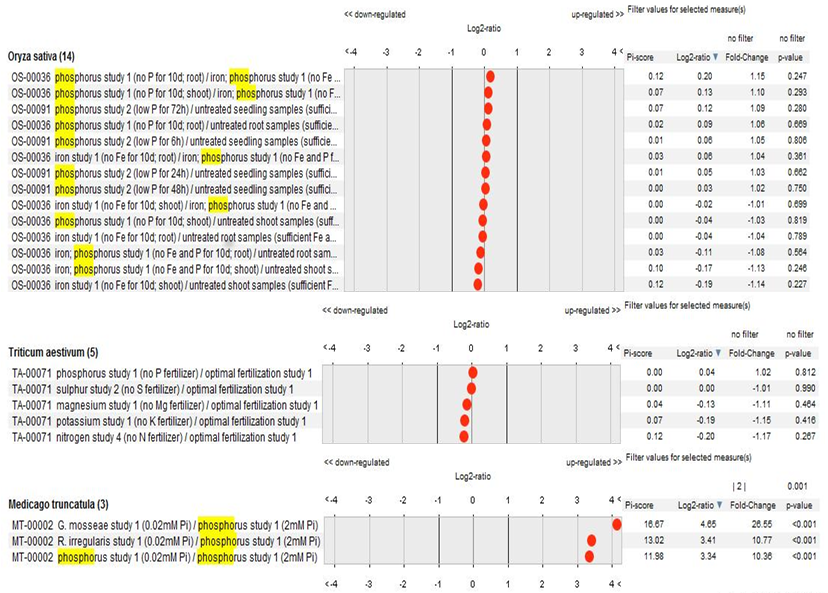

Supplement: S8 Fig — (TIF) [file pone.0213531.s008.tif]
