# Supplementary material for: A study of CCD8 genes/proteins in seven monocots and eight dicots
Source: PLoS One. 2019 Mar 12;14(3):e0213531. doi: 10.1371/journal.pone.0213531 (PMC6413960; doi:10.1371/journal.pone.0213531)
Supplement: S1 Table — Details of the positions of exons (upper row) and introns (lower row) of CCD8 structural gene sequences (in bp) in 15 different species. The position of first exon is marked from translation start site. (DOCX) [file pone.0213531.s009.docx]

**Supplementary material**

**A study of CCD8 genes/proteins in seven monocots and eight dicots**

Ritu Batra^1^, Priyanka Agarwal^1^, Sandhya Tyagi^2^, Dinesh Kumar Saini^1^, Vikas Kumar^1^, Anuj Kumar^3^, Sanjay Kumar^4^, Harindra Singh Balyan^1^, Renu Pandey^2^

and Pushpendra Kumar Gupta^1^*

*Correspondence:

Pushpendra Kumar Gupta

email: [pkgupta36@gmail.com](mailto:pkgupta36@gmail.com)

**S1 Table.** Details of the positions of exons (upper row) and introns (lower row) of CCD8 structural gene sequences (in bp) in 15 different species. The position of first exon is marked from translation start site.

| Species | 1 | 2 | | | | | 3 | | 4 |
| --- | --- | --- | --- | --- | --- | --- | --- | --- | --- |
| *Z.mays* | 133-462 | 1575-2512 | | | | | 2607-2899 | | 3025-3182 |
|  | 463-1574 | 2513-2606 | | | | | 2900-3024 | | - |
| *T.aestivum* sub-genome A | 172-474 | 1707-2450 | | | 2569-2762 | | 2859-3151 | | 3242-3390 |
|  | 475-1706 | 2451-2568 | | | 2763-2858 | | 3152-3241 | | - |
| *T.aestivum* sub-genome B | 111-413 | 1657-2400 | | | 2514-2707 | | 2806-3098 | | 3183-3331 |
|  | 414-1656 | 2401-2513 | | | 2708-2805 | | 3099-3182 | | - |
| *T.aestivum* sub-genome D | 109-411 | 1637-2380 | | | 2492-2685 | | 2785-3077 | | 3165-3313 |
|  | 412-1636 | 2381-2491 | | | 2686-2784 | | 3078-3164 | | - |
| *T. urartu* | 1-156 | 374-550 | 592-750 | 876-1151 | 1267-1460 | | 1557-1849 | | 1940-2088 |
|  | 157-373 | 551-591 | 751-875 | 1152-1266 | 1461-1556 | | 1850-1939 | |  |
| *Ae. tauschi* | 1-144 | 1496-2239 | | | 2351-2544 | | 2643-2936 | | 3024-3172 |
|  | 145-1495 | 2240-2350 | | | 2545-2643 | | 2937-3023 | | - |
| *O. sativa* | 1-321 | 1416-2159 | | | 2266-2459 | | 2559-2851 | | 2952-3109 |
|  | 322-1415 | 2160-2265 | | | 2460-2558 | | 2852-2951 | | - |
| *B. distachyon* | 1-333 | 1570-2313 | | | 2411-2897 | | | | 3002-3150 |
|  | 334-1569 | 2314-2410 | | | 2898-3001 | | | | - |
| *S. bicolor* | 128-478 | 1950-2887 | | | 2974-3266 | | | | 3388-3545 |
|  | 479-1949 | 2888-2973 | | | 3267-3387 | | | | - |
| *A. thaliana* | 121-435 | 1177-1938 | | 2201-2397 | 2485-2674 | 2753-2855 | | | 2927-3072 |
|  | 436-1176 | 1939-2200 | | 2398-2484 | 2675-2752 | 2856-2926 | | | - |
| *G. max* | 136-450 | 1535-2278 | | 2672-2865 | 2983-3172 | 3323-3425 | | | 3524-3669 |
|  | 451-1534 | 2279-2671 | | 2866-2982 | 3173-3322 | 3426-3523 | | | - |
| *V. vinifera* | 1-264 | 880-1623 | | 1710-1903 | 2163-2352 | 2434-2682 | | | |
|  | 265-879 | 1624-1709 | | 1904-2162 | 2353-2433 | - | | | |
| *S. lycopersicon* | 44-340 | 1223-1966 | | 2046-2239 | 2310-2499 | 2566-2668 | | 2740-2885 | |
|  | 341-1222 | 1967-2045 | | 2240-2309 | 2500-2565 | 2669-2739 | | - | |
| *T. cacao* | 67-369 | 1129-1872 | | 1961-2154 | 2679-2868 | 2991-3093 | | 3230-3375 | |
|  | 370-1128 | 1873-1960 | | 2155-2678 | 2869-2990 | 3094-3229 | | - | |
| *P. trichocarpa* | 1-297 | 1347-2090 | | 2203-2396 | 2708-2897 | 3092-3194 | | 3838-3983 | |
|  | 298-1346 | 2091-2202 | | 2397-2707 | 2898-3091 | 3195-3837 | | - | |
| *P. persica* | * | 1136-1798 | | 1922-2115 | 2260-2449 | 2537-2639 | | 2743-2888 | |
|  | - | 1799-1921 | | 2116-2259 | 2450-2536 | 2640-2742 | | - | |
| *M. truncatula* | 67-387 | 1381-2124 | | 2222-2415 | 2532-2721 | 2813-2915 | | 3001-3146 | |
|  | 388-1380 | 2125-2221 | | 2416-2531 | 2722-2812 | 2916-3000 | | - | |

A star (*) in this column means absence of exons and a dash (-) in this column means absence of introns
