# Supplementary material for: A study of CCD8 genes/proteins in seven monocots and eight dicots
Source: PLoS One. 2019 Mar 12;14(3):e0213531. doi: 10.1371/journal.pone.0213531 (PMC6413960; doi:10.1371/journal.pone.0213531)
Supplement: S2 Table — (DOCX) [file pone.0213531.s010.docx]

**Supplementary material**

**A study of CCD8 genes/proteins in seven monocots and eight dicots**

Ritu Batra^1^, Priyanka Agarwal^1^, Sandhya Tyagi^2^, Dinesh Kumar Saini^1^, Vikas Kumar^1^, Anuj Kumar^3^, Sanjay Kumar^4^, Harindra Singh Balyan^1^, Renu Pandey^2^

and Pushpendra Kumar Gupta^1^*

*Correspondence:

Pushpendra Kumar Gupta

email: [pkgupta36@gmail.com](mailto:pkgupta36@gmail.com)

**S2 Table**. Per cent similarity of exons in CCD8 genes of 14 different species with respect to *Z. mays*.

| Species | Exon 1 | Exon 2 | Exon 3 | Exon 4 | Exon 5 | Exon 6 |
| --- | --- | --- | --- | --- | --- | --- |
| *Z. mays* | 100 | 100 | 100 | 100 | 100 | 100 |
| *T.aestivum* sub-genome A | 70.7 | 89.52 | 88.66 | 92.15 | 93.2 | 85.23 |
| *T.aestivum* sub-genome B | 69.96 | 88.84 | 87.63 | 92.49 | 94.17 | 85.91 |
| *T.aestivum* sub-genome D | 69.96 | 89.25 | 89.18 | 91.81 | 95.15 | 85.23 |
| *T. urartu* | 40.78 | 87.1 | 90.21 | 91.47 | 92.23 | 88.24 |
| *Ae. tauschi* | 65.49 | 89.25 | 89.18 | 91.81 | 95.15 | 85.23 |
| *O. sativa* | 75.61 | 91.26 | 93.3 | 91.13 | 90.29 | 84.18 |
| *B. distachyon* | 70.96 | 88.71 | 87.23 | 90.78 | 91.26 | 85.91 |
| *S. bicolor* | 85.15 | 96.16 | 96.16 | 97.95 | 99.03 | 94.3 |
| *A. thaliana* | 37.1 | 63.84 | 70.1 | 68.95 | 67.96 | 57.23 |
| *G. max* | 41.01 | 70.43 | 69.59 | 69.47 | 67.96 | 60.96 |
| *V. vinifera* | 46.7 | 70.7 | 71.13 | 73.68 | 70.87 | 65.75 |
| *S.lycopersicum* | 39.02 | 62.9 | 72.68 | 67.37 | 69.9 | 57.53 |
| *T. cacao* | 41.48 | 65.32 | 73.2 | 76.32 | 66.99 | 58.22 |
| *P. trichocarpa* | 39.62 | 67.74 | 69.07 | 72.11 | 72.82 | 59.59 |
| *P. persica* | * | 66.67 | 69.07 | 67.02 | 68.93 | 60.96 |
| *M. truncatula* | 41.55 | 66.4 | 71.13 | 68.95 | 65.05 | 57.53 |

A star (*) in this column means absence of exons
