# Supplementary material for: A study of CCD8 genes/proteins in seven monocots and eight dicots
Source: PLoS One. 2019 Mar 12;14(3):e0213531. doi: 10.1371/journal.pone.0213531 (PMC6413960; doi:10.1371/journal.pone.0213531)
Supplement: S3 Table — Values of non-synonymous substitutions (Ka; upper row) and synonymous substitutions (Ks; lower row) in CCD8 genes of seven monocots (including homoeologues on group 3 chromosomes of wheat). (DOCX) [file pone.0213531.s011.docx]

**Supplementary material**

**A study of CCD8 genes/proteins in seven monocots and eight dicots**

Ritu Batra^1^, Priyanka Agarwal^1^, Sandhya Tyagi^2^, Dinesh Kumar Saini^1^, Vikas Kumar^1^, Anuj Kumar^3^, Sanjay Kumar^4^, Harindra Singh Balyan^1^, Renu Pandey^2^

and Pushpendra Kumar Gupta^1^*

*Correspondence:

Pushpendra Kumar Gupta

email: [pkgupta36@gmail.com](mailto:pkgupta36@gmail.com)

**S3 Table.** Values of non-synonymous substitutions (Ka; upper row) and synonymous substitutions (Ks; lower row) in CCD8 genes of seven monocots (including homoeologues on group 3 chromosomes of wheat).

| Species |  |  |  |  |  |  |  |  |  | Average | Ka/ks |
| --- | --- | --- | --- | --- | --- | --- | --- | --- | --- | --- | --- |
| *Z.mays* | 0.000 |  |  |  |  |  |  |  |  |  |  |
|  | 0.000 |  |  |  |  |  |  |  |  |  |  |
| *T.aestivum* sub-genome A | 0.147 | 0 |  |  |  |  |  |  |  |  |  |
|  | 0.043 | 0 |  |  |  |  |  |  |  |  |  |
| *T.aestivum* sub-genome B | 0.153 | 0.032 | 0 |  |  |  |  |  |  |  |  |
|  | 0.039 | 0.012 | 0 |  |  |  |  |  |  |  |  |
| *T.aestivum* sub-genome D | 0.150 | 0.024 | 0.034 | 0 |  |  |  |  |  |  |  |
|  | 0.040 | 0.002 | 0.007 | 0 |  |  |  |  |  |  |  |
| *T. urartu* | 0.166 | 0.074 | 0.080 | 0.080 | 0 |  |  |  |  |  |  |
|  | 0.053 | 0.035 | 0.027 | 0.031 | 0 |  |  |  |  |  |  |
| *Ae. tauschi* | 0.146 | 0.023 | 0.033 | 0.000 | 0.075 | 0 |  |  |  |  |  |
|  | 0.037 | 0.002 | 0.007 | 0.000 | 0.028 | 0 |  |  |  |  |  |
| *O. sativa* | 0.109 | 0.100 | 0.107 | 0.101 | 0.109 | 0.097 | 0 |  |  |  |  |
|  | 0.050 | 0.040 | 0.048 | 0.043 | 0.057 | 0.040 | 0 |  |  |  |  |
| *B. distachyon* | 0.155 | 0.122 | 0.128 | 0.124 | 0.149 | 0.118 | 0.124 | 0 |  |  |  |
|  | 0.040 | 0.028 | 0.030 | 0.026 | 0.044 | 0.024 | 0.041 | 0 |  |  |  |
| *S. bicolor* | 0.051 | 0.140 | 0.149 | 0.149 | 0.150 | 0.144 | 0.104 | 0.156 | 0 | 0.084 |  |
|  | 0.002 | 0.039 | 0.035 | 0.037 | 0.049 | 0.024 | 0.047 | 0.040 | 0 | 0.026 | 3.23 |
