# Supplementary material for: A study of CCD8 genes/proteins in seven monocots and eight dicots
Source: PLoS One. 2019 Mar 12;14(3):e0213531. doi: 10.1371/journal.pone.0213531 (PMC6413960; doi:10.1371/journal.pone.0213531)
Supplement: S4 Table — Values of non-synonymous substitutions (Ka; upper row) and synonymous substitutions (Ks; lower row) in CCD8 genes of eight dicots. (DOCX) [file pone.0213531.s012.docx]

**Supplementary material**

**A study of CCD8 genes/proteins in seven monocots and eight dicots**

Ritu Batra^1^, Priyanka Agarwal^1^, Sandhya Tyagi^2^, Dinesh Kumar Saini^1^, Vikas Kumar^1^, Anuj Kumar^3^, Sanjay Kumar^4^, Harindra Singh Balyan^1^, Renu Pandey^2^

and Pushpendra Kumar Gupta^1^*

*Correspondence:

Pushpendra Kumar Gupta

email: [pkgupta36@gmail.com](mailto:pkgupta36@gmail.com)

**S4 Table**. Values of non-synonymous substitutions (Ka; upper row) and synonymous substitutions (Ks; lower row) in CCD8 genes of eight dicots.

| Species |  |  |  |  |  |  |  |  | Average | Ka/Ks |
| --- | --- | --- | --- | --- | --- | --- | --- | --- | --- | --- |
| *A. thaliana* | 0 |  |  |  |  |  |  |  |  |  |
|  | 0.000 |  |  |  |  |  |  |  |  |  |
| *G. max* | 0.462 | 0 |  |  |  |  |  |  |  |  |
|  | 0.162 | 0 |  |  |  |  |  |  |  |  |
| *V. vinifera* | 0.448 | 0.291 | 0 |  |  |  |  |  |  |  |
|  | 0.174 | 0.061 | 0 |  |  |  |  |  |  |  |
| *S .lycopersicum* | 0.472 | 0.357 | 0.319 | 0 |  |  |  |  |  |  |
|  | 0.206 | 0.080 | 0.097 | 0 |  |  |  |  |  |  |
| *T. cacao* | 0.424 | 0.325 | 0.325 | 0.337 | 0 |  |  |  |  |  |
|  | 0.190 | 0.087 | 0.078 | 0.114 | 0 |  |  |  |  |  |
| *P. trichocarpa* | 0.473 | 0.295 | 0.294 | 0.312 | 0.280 | 0 |  |  |  |  |
|  | 0.168 | 0.053 | 0.050 | 0.077 | 0.083 | 0 |  |  |  |  |
| *P. persica* | 0.414 | 0.270 | 0.288 | 0.334 | 0.327 | 0.261 | 0 |  |  |  |
|  | 0.168 | 0.070 | 0.071 | 0.075 | 0.079 | 0.059 | 0 |  |  |  |
| *M. truncatula* | 0.497 | 0.216 | 0.322 | 0.333 | 0.318 | 0.297 | 0.293 | 0 | 0.266 |  |
|  | 0.197 | 0.050 | 0.071 | 0.077 | 0.104 | 0.055 | 0.091 | 0 | 0.079 | 3.37 |
