# Supplementary material for: A study of CCD8 genes/proteins in seven monocots and eight dicots
Source: PLoS One. 2019 Mar 12;14(3):e0213531. doi: 10.1371/journal.pone.0213531 (PMC6413960; doi:10.1371/journal.pone.0213531)
Supplement: S5 Table — (DOCX) [file pone.0213531.s013.docx]

**Supplementary material**

**A study of CCD8 genes/proteins in seven monocots and eight dicots**

Ritu Batra^1^, Priyanka Agarwal^1^, Sandhya Tyagi^2^, Dinesh Kumar Saini^1^, Vikas Kumar^1^, Anuj Kumar^3^, Sanjay Kumar^4^, Harindra Singh Balyan^1^, Renu Pandey^2^

and Pushpendra Kumar Gupta^1^*

*Correspondence:

Pushpendra Kumar Gupta

email: [pkgupta36@gmail.com](mailto:pkgupta36@gmail.com)

**S5 Table**. Details of 31 genes (including the CCD8 gene of *Z. mays* at position 16) used for synteny and collinearity analysis.

| **S.No** | **Genomicus gene id** | **Ensembl Plants gene id** | **Description of the gene** | **Chromosome number and coordinates** |
| --- | --- | --- | --- | --- |
| 1 | *GRMZM2G103666* | *Zm00001d043461* | ZCN12 | 3:200703155-200704705 |
| 2 | *GRMZM2G045366* | *Zm00001d043459* | MAP kinase kinase kinase47 | 3:200575782-200580113 |
| 3 | *GRMZM2G045215* | *Zm00001d043458* | GDSL esterase/lipase | 3:200565289-200567523 |
| 4 | *GRMZM2G314582* | *Zm00001d043456* | n/a | 3:200488674-200489138 |
| 5 | *GRMZM2G010936* | gene not found | n/a | 3:200478527-200479283 |
| 6 | *GRMZM2G010804* | *Zm00001d043454* | Whole genome shotgun sequence of line PN40024 scaffold_142.assembly12x | 3:200474841-200475905 |
| 7 | *GRMZM2G010762* | *Zm00001d043453* | Early nodulin-like protein 3 | 3:200465188-200466146 |
| 8 | *GRMZM2G314546* | *Zm00001d043452* | homeobox3 | [3:200451086-200464251](https://plants.ensembl.org/Zea_mays/Location/View?r=3:200448453-200466884;g=Zm00001d043452;db=) |
| 9 | *GRMZM2G010306* | *Zm00001d043451* | Dynamin-related protein 5A | 3:200445235-200450462 |
| 10 | *GRMZM2G314520* | *Zm00001d043450* | DNA-binding WRKY | 3:200441483-200442238 |
| 11 | *GRMZM2G105425* | *Zm00001d043449* | RNA-binding S4 domain-containing protein | 3:200412705-200420724 |
| 12 | *GRMZM2G317285* | *Zm00001d043446* | Pentatricopeptide repeat-containing protein mitochondrial | 3:200383769-200385995 |
| 13 | *GRMZM2G021704* | *Zm00001d043445* | Dihydroorotase | 3:200377050-200383360 |
| 14 | *GRMZM2G069694* | *Zm00001d043444* | plant-specific domain TIGR01615 family protein expressed | 3:200352833-200354707 |
| 15 | *GRMZM2G140201* | *Zm00001d043443* | Mannan endo-14-beta-mannosidase 2 | 3:200300085-200302486 |
| **16** | ***GRMZM2G446858*** | [**Zm00001d043442_T001**](http://plants.ensembl.org/Zea_mays/Transcript/Summary?db=core;g=Zm00001d043442;r=3:200059008-200062529;t=Zm00001d043442_T001) | **carotenoid cleavage dioxygenase8** | [**3: 200,059,008-200,062,529**](http://plants.ensembl.org/Zea_mays/Location/View?db=core;g=Zm00001d043442;r=3:200059008-200062529;t=Zm00001d043442_T001) |
| 17 | *GRMZM2G028159* | *Zm00001d043421* | n/a | 3:199156004-199157305 |
| 18 | *GRMZM2G028234* | *Zm00001d043422* | RING-H2 finger protein ATL2K | 3:199163496-199164221 |
| 19 | *GRMZM2G194106* | gene not found | n/a |  |
| 20 | *GRMZM2G309660* | *Zm00001d043425* | n/a | 3:199386458-199395287 |
| 21 | *GRMZM2G352234* | *Zm00001d043426* | Pentatricopeptide repeat-containing protein | 3:199479040-199481034 |
| 22 | *GRMZM2G052776* | *Zm00001d043427* | n/a | 3:199485601-199485861 |
| 23 | *GRMZM2G022856* | *Zm00001d043428* | Callose synthase 11 | 3:199525342-199532354 |
| 24 | *GRMZM2G167174* | *Zm00001d043429* | n/a | 3:199571249-199573965 |
| 25 | *AC210193.4_FG002* | *Zm00001d043430* | n/a | 3:199604217-199605056 |
| 26 | *GRMZM2G056120* | *Zm00001d043431* | Auxin response factor 11 | 3:199611426-199617608 |
| 27 | *GRMZM2G055857* | *Zm00001d043432* | GroES-like zinc-binding alcohol dehydrogenase family protein | 3:199636305-199641922 |
| 28 | *GRMZM2G443814* | *Zm00001d043434* | Zinc finger CCCH domain-containing protein 19 | 3:199733893-199758701 |
| 29 | *GRMZM2G409473* | gene not found | n/a |  |
| 30 | *GRMZM2G409430* | *Zm00001d043439* | Nuclear pore complex protein NUP85 | 3:199908397-199919731 |
| 31 | *GRMZM2G154165* | *Zm00001d043441* | Dolichyl-diphosphooligosaccharide--protein glycosyltransferase subunit STT3A | 3:199979127-199987549 |
