# Supplementary material for: A study of CCD8 genes/proteins in seven monocots and eight dicots
Source: PLoS One. 2019 Mar 12;14(3):e0213531. doi: 10.1371/journal.pone.0213531 (PMC6413960; doi:10.1371/journal.pone.0213531)
Supplement: S6 Table — (DOCX) [file pone.0213531.s014.docx]

**Supplementary material**

**A study of CCD8 genes/proteins in seven monocots and eight dicots**

Ritu Batra^1^, Priyanka Agarwal^1^, Sandhya Tyagi^2^, Dinesh Kumar Saini^1^, Vikas Kumar^1^, Anuj Kumar^3^, Sanjay Kumar^4^, Harindra Singh Balyan^1^, Renu Pandey^2^

and Pushpendra Kumar Gupta^1^*

*Correspondence:

Pushpendra Kumar Gupta

email: [pkgupta36@gmail.com](mailto:pkgupta36@gmail.com)

**S6 Table.** Simple sequence repeats (SSRs) and retro-elements identified in CCD8 genes belonging to 12 species.

| Species | Position | Repeat sequence | Number |
| --- | --- | --- | --- |
| *Z. mays* | 42-80;420-463 | (GCTA)n; (GGAGCT)n | 2 |
| *T.aestivum* sub-genome A | 208-250;345-379 | (GCCG)n; (CGGCG)n | 2 |
| *T.aestivum* sub-genome B | 147-189 | (GCCG)n | 1 |
| *T.aestivum* sub-genome D | 145-187 | (GCCG)n | 1 |
| *T. urartu* | 2558-2598 | (TTATATT)n | 1 |
| *Ae. tauschi* | 37-79 | (GCCG)n | 1 |
| *B. distachyon* | 140-164 | (CGG)n | 1 |
| *S. bicolor* | 23-48;436-479;1923-1944;2893-2928 | (GCTA)n; (GGAGCT)n; (TTAA)n; (GCT)n | 4 |
| *A. thaliana* | 680-706 | (A)n | 1 |
| *G. max* | 1386-1442;3181-3225 | (AATTAT)n; (TA)n | 2 |
| *T. cacao* | 695-738; 2162-2204; 2303-2325 | (TT)n; (AT)n; (TTA)n | 3 |
| *P. trichocarpa* | 1109-1280; 3278-3304; 3605-3744 | (AG)n | 1; 1; 1 |
| *P. persica* | 2647-2675; 2676-2698 | (TTAT)n; (TA)n | 2 |
| *M. truncatula* | 950-987; 2185-2219 | (AATT)n; (TTGATT)n | 2 |
