# Supplementary material for: A study of CCD8 genes/proteins in seven monocots and eight dicots
Source: PLoS One. 2019 Mar 12;14(3):e0213531. doi: 10.1371/journal.pone.0213531 (PMC6413960; doi:10.1371/journal.pone.0213531)
Supplement: S7 Table — (DOCX) [file pone.0213531.s015.docx]

**Supplementary material**

**A study of CCD8 genes/proteins in seven monocots and eight dicots**

Ritu Batra^1^, Priyanka Agarwal^1^, Sandhya Tyagi^2^, Dinesh Kumar Saini^1^, Vikas Kumar^1^, Anuj Kumar^3^, Sanjay Kumar^4^, Harindra Singh Balyan^1^, Renu Pandey^2^

and Pushpendra Kumar Gupta^1^*

*Correspondence:

Pushpendra Kumar Gupta

email: [pkgupta36@gmail.com](mailto:pkgupta36@gmail.com)

**S7 Table.** Primary protein sequence analyses of CCD8 proteins of 15 selected species.

| Species | Mol wt. | pI | Negitively charged AA | Positively charged AA | Instability Index | Aliphatic Index | Gravy |
| --- | --- | --- | --- | --- | --- | --- | --- |
| Z. mays | 62.41 | 6.53 | 66 | 62 | 42.03 | 76.05 | -0.252 |
| *T.aestivum* sub-genome A | 61.35 | 6.24 | 71 | 65 | 40.72 | 77.68 | -0.292 |
| *T.aestivum* sub-genome B | 61.3 | 6.05 | 71 | 63 | 39.34 | 78.38 | -0.277 |
| *T.aestivum* sub-genome D | 61.25 | 6.14 | 71 | 64 | 40.4 | 78.73 | -0.27 |
| *T. urartu* | 51.48 | 5.87 | 63 | 53 | 45.64 | 76.45 | -0.349 |
| *Ae. tauschi* | 55.51 | 5.96 | 64 | 55 | 37.59 | 77.73 | -0.252 |
| *O. sativa* | 61.98 | 6.51 | 66 | 62 | 42.99 | 75.76 | -0.292 |
| *B. distachyon* | 62.39 | 5.85 | 72 | 62 | 43.81 | 75.32 | -0.285 |
| *S. bicolor* | 63.08 | 6.61 | 65 | 62 | 40.58 | 75.13 | -0.262 |
| *A. thaliana* | 63.96 | 6.65 | 70 | 66 | 30.66 | 81.89 | -0.326 |
| *G. max* | 62.98 | 6.22 | 68 | 60 | 42.22 | 80.87 | -0.321 |
| *V. vinifera* | 60.53 | 6.99 | 65 | 64 | 31.91 | 79.8 | -0.324 |
| *S. lycopersicum* | 62.07 | 6.37 | 72 | 67 | 29.12 | 82.78 | -0.313 |
| *T. cacao* | 62.51 | 6.69 | 69 | 66 | 37.46 | 80.39 | -0.366 |
| *P. trichocarpa* | 61.89 | 5.75 | 73 | 61 | 32.72 | 78.06 | -0.313 |
| *P. persica* | 47.66 | 5.75 | 54 | 44 | 30.99 | 83.29 | -0.214 |
| *M. truncatula* | 63.12 | 6.11 | 68 | 59 | 38.5 | 78.83 | -0.366 |
