# Supplementary material for: A study of CCD8 genes/proteins in seven monocots and eight dicots
Source: PLoS One. 2019 Mar 12;14(3):e0213531. doi: 10.1371/journal.pone.0213531 (PMC6413960; doi:10.1371/journal.pone.0213531)
Supplement: S8 Table — (DOCX) [file pone.0213531.s016.docx]

**Supplementary material**

**A study of CCD8 genes/proteins in seven monocots and eight dicots**

Ritu Batra^1^, Priyanka Agarwal^1^, Sandhya Tyagi^2^, Dinesh Kumar Saini^1^, Vikas Kumar^1^, Anuj Kumar^3^, Sanjay Kumar^4^, Harindra Singh Balyan^1^, Renu Pandey^2^

and Pushpendra Kumar Gupta^1^*

*Correspondence:

Pushpendra Kumar Gupta

email: [pkgupta36@gmail.com](mailto:pkgupta36@gmail.com)

**S8 Table.** Analysis of secondary structure of CCD8 proteins of selected 15 species.

| Species | α-helix | Extended strand | β-turn | Random coil |
| --- | --- | --- | --- | --- |
| *Z. mays* | 23.95 | 24.48 | 11.19 | 40.38 |
| *T.aestivum* sub-genome A | 28.04 | 23.75 | 12.14 | 36.07 |
| *T.aestivum* sub-genome B | 28.21 | 23.04 | 11.79 | 36.96 |
| *T.aestivum* sub-genome D | 28.75 | 22.86 | 11.07 | 37.32 |
| *T. urartu* | 19.06 | 22.27 | 6.64 | 52.03 |
| *Ae. tauschi* | 28.01 | 23.67 | 11.83 | 36.49 |
| *O. sativa* | 27.24 | 22.67 | 11.42 | 38.66 |
| *B. distachyon* | 30 | 22.28 | 13.1 | 34.56 |
| *S. bicolor* | 34.7 | 23.49 | 10.88 | 40.93 |
| *A. thaliana* | 27.37 | 25.79 | 11.23 | 35.61 |
| *G. max* | 19.89 | 27 | 11.19 | 41.92 |
| *V. vinifera* | 30.04 | 22.34 | 11.36 | 36.26 |
| *S. lycopersicum* | 27.11 | 25.67 | 11.85 | 35.37 |
| *T. cacao* | 22.18 | 26.83 | 12.16 | 38.82 |
| *P. trichocarpa* | 26.93 | 23.7 | 14.18 | 35.19 |
| *P. persica* | 23.2 | 26.91 | 11.83 | 38.05 |
| *M. truncatula* | 19.29 | 29.56 | 11.86 | 39.29 |
