# Supplementary material for: A study of CCD8 genes/proteins in seven monocots and eight dicots
Source: PLoS One. 2019 Mar 12;14(3):e0213531. doi: 10.1371/journal.pone.0213531 (PMC6413960; doi:10.1371/journal.pone.0213531)
Supplement: S9 Table — (DOCX) [file pone.0213531.s017.docx]

**Supplementary material**

**A study of CCD8 genes/proteins in seven monocots and eight dicots**

Ritu Batra^1^, Priyanka Agarwal^1^, Sandhya Tyagi^2^, Dinesh Kumar Saini^1^, Vikas Kumar^1^, Anuj Kumar^3^, Sanjay Kumar^4^, Harindra Singh Balyan^1^, Renu Pandey^2^

and Pushpendra Kumar Gupta^1^*

*Correspondence:

Pushpendra Kumar Gupta

email: [pkgupta36@gmail.com](mailto:pkgupta36@gmail.com)

**S9 Table.** Predicted values of different parameters for the motifs identified in CCD8 proteins using MEME suite.

| **Motif** | **E value** | **Site-count** | **Width** | **Novelty** | **Consensus sequence of motif** |
| --- | --- | --- | --- | --- | --- |
| 1 | 7.7e-729 | 17 | 50 | No | HGRGMDMCSINPAHLGKEYRYAYACGARRPCNFPNTLTKIDLVEKTAKNW |
| 2 | 1.5e-726 | 17 | 50 | No | WVHSFPVTENYVVVPEMPLRYCAANLLRAEPTPLYKFQWHLESGSYMHVM |
| 3 | 2.2e-555 | 17 | 35 | Yes | DEEGRVTAIIADCCEHNADTSILDNLRLHNLRAFT |
| 4 | 6.7e-645 | 17 | 50 | No | EEDDGVAISMVSAKDGSGYALVLDAKSFKEIARAKFPYGLPYGLHCCWVP |
| 5 | 5.5e-618 | 17 | 50 | No | FSGNSLTDNSNTGVVRLGDGRVLCLTETVKGSIVVDPDTLDTVSKFEYED |
| 6 | 2.7e-329 | 17 | 29 | Yes | DVLTDARVGRFRIPLDGSPFGELEAALDP |
| 7 | 1.3e-275 | 17 | 21 | Yes | IVASVEVPPFVTFHFINAYEE |
| 8 | 9.6e-156 | 17 | 15 | Yes | EGAVPSEPYFVARPG |
| 9 | 1.0e-599 | 16 | 50 | No | TYLRNGPGLWDLGDYGFRHLFDGYATLVRVSFRDGRAVGAHRQIESEAYK |
| 10 | 3.1e-575 | 16 | 50 | No | LGGLIHSAHPIVTDTEFWTLIPDLIRPGYVVARMDAGSNERQFVGRVDCR |
| 11 | 1.7e-257 | 15 | 29 | Yes | RRHGKVCYREFSEVPKPDSFKSFVGQLAS |
| 12 | 1.4e-250 | 14 | 29 | Yes | ELVAWKSIRQERWEGALEVEGKLPTWLDG |
