# Supplementary material for: A study of CCD8 genes/proteins in seven monocots and eight dicots
Source: PLoS One. 2019 Mar 12;14(3):e0213531. doi: 10.1371/journal.pone.0213531 (PMC6413960; doi:10.1371/journal.pone.0213531)
Supplement: S10 Table — (DOCX) [file pone.0213531.s018.docx]

**Supplementary material**

**A study of CCD8 genes/proteins in seven monocots and eight dicots**

Ritu Batra^1^, Priyanka Agarwal^1^, Sandhya Tyagi^2^, Dinesh Kumar Saini^1^, Vikas Kumar^1^, Anuj Kumar^3^, Sanjay Kumar^4^, Harindra Singh Balyan^1^, Renu Pandey^2^

and Pushpendra Kumar Gupta^1^*

*Correspondence:

Pushpendra Kumar Gupta

email: [pkgupta36@gmail.com](mailto:pkgupta36@gmail.com)

**S10 Table.** Details of 3D structures of CCD8 proteins (using Swiss-model) and their quality assessment parameters in selected 15 species obtained using SAVES and structure assessment tool of Swiss-Model.

| Species | % identity | GMQE | Residues in favoured region | Residues in allowed region | Residues in outlier region | ERRAT  (%) | Verify 3D(3D-1D Profile) (%) | D-fire |
| --- | --- | --- | --- | --- | --- | --- | --- | --- |
| *Z. mays* | 26.71 | 0.53 | 73.3 | 17.5 | 9.1 | 77.40 | 76.4 | -714.61 |
| *T.aestivum* sub-genome A | 30.41 | 0.53 | 71.5 | 16.5 | 12 | 72.23 | 92.86 | -716.77 |
| *T.aestivum* sub-genome B | 30.41 | 0.52 | 75.3 | 15.4 | 9.3 | 74.18 | 86.07 | -719.35 |
| *T.aestivum* sub-genome D | 29.73 | 0.55 | 74.7 | 17 | 8.2 | 75.91 | 86.96 | -723.82 |
| *T. urartu* | 23.9 | 0.40 | 66.8 | 20.2 | 13 | 74.78 | 75.48 | -653.16 |
| *Ae. tauschi* | 28.55 | 0.54 | 71.5 | 18.8 | 9.7 | 76.75 | 84.42 | -654.75 |
| *O. sativa* | 31.22 | 0.53 | 72.1 | 17.1 | 10.8 | 76.92 | 79.44 | -701.9 |
| *B. distachyon* | 30.29 | 0.47 | 67.4 | 21 | 11.6 | 76.61 | 85.79 | -704.31 |
| *S. bicolor* | 30.17 | 0.47 | 72.4 | 18 | 9.7 | 73.47 | 80.38 | -742.15 |
| *A. thaliana* | 31.21 | 0.50 | 71.5 | 19 | 9.5 | 76.83 | 71.93 | -733.61 |
| *G. max* | 28.79 | 0.54 | 72.9 | 17.5 | 9.6 | 80.14 | 88.63 | -752.2 |
| *V. vinifera* | 29.84 | 0.51 | 73.5 | 16.2 | 10.3 | 89.96 | 83.88 | -720.05 |
| *S. lycopersicum* | 28.94 | 0.55 | 75.5 | 16.2 | 8.3 | 79.78 | 80.25 | -744.62 |
| *T. cacao* | 27.55 | 0.60 | 69.3 | 18.5 | 12.2 | 80 | 79.43 | - |
| *P. trichocarpa* | 28.11 | 0.54 | 67.6 | 20.4 | 12.1 | 79.02 | 84.38 | - |
| *P. persica* | 32.80 | 0.53 | 78.8 | 16.6 | 4.7 | 77.43 | 90.26 | -544.01 |
| *M. truncatula* | 29.58 | 0.51 | 68.7 | 20.1 | 11.2 | 75.09 | 81.24 | - |
