# Supplementary material for: A study of CCD8 genes/proteins in seven monocots and eight dicots
Source: PLoS One. 2019 Mar 12;14(3):e0213531. doi: 10.1371/journal.pone.0213531 (PMC6413960; doi:10.1371/journal.pone.0213531)
Supplement: S11 Table — (DOCX) [file pone.0213531.s019.docx]

**Supplementary material**

**A study of CCD8 genes/proteins in seven monocots and eight dicots**

Ritu Batra^1^, Priyanka Agarwal^1^, Sandhya Tyagi^2^, Dinesh Kumar Saini^1^, Vikas Kumar^1^, Anuj Kumar^3^, Sanjay Kumar^4^, Harindra Singh Balyan^1^, Renu Pandey^2^

and Pushpendra Kumar Gupta^1^*

*Correspondence:

Pushpendra Kumar Gupta

email: [pkgupta36@gmail.com](mailto:pkgupta36@gmail.com)

**S11 Table.** Predicted values of different parameters obtained after superimposition of 3D protein structures of CCD8 of selected 14 species over 3D protein structure of CCD8 of maize.

| *Species* | RMSD | Similarity | Identity |
| --- | --- | --- | --- |
| *Z. mays* | 0 | 100 | 100 |
| *T.aestivum* sub-genome A | 3.11 | 85.19 | 78.33 |
| *T.aestivum* sub-genome B | 3.04 | 84.19 | 77.57 |
| *T.aestivum* sub-genome D | 3.26 | 83.58 | 77.12 |
| *T. urartu* | 3.03 | 50.98 | 44.12 |
| *Ae. tauschi* | 3.17 | 84.51 | 78.43 |
| *O. sativa* | 3.06 | 82.18 | 75.78 |
| *B. distachyon* | 2.99 | 84.31 | 77.36 |
| *S. bicolor* | 2.11 | 72.71 | 70.38 |
| *A. thaliana* | 1.72 | 69.24 | 55.62 |
| *G. max* | 3.02 | 69.03 | 57.79 |
| *V. vinifera* | 3.1 | 80.44 | 68.27 |
| *S. lycopersicum* | 3.01 | 57.47 | 68.06 |
| *T. cacao* | 3.05 | 75.31 | 62.52 |
| *P. trichocarpa* | 2.98 | 74.96 | 64.04 |
| *P. persica* | 3.02 | 76.13 | 65.54 |
| *M.truncatula* | 3.08 | 76.56 | 65.1 |
