# Supplementary material for: A study of CCD8 genes/proteins in seven monocots and eight dicots
Source: PLoS One. 2019 Mar 12;14(3):e0213531. doi: 10.1371/journal.pone.0213531 (PMC6413960; doi:10.1371/journal.pone.0213531)
Supplement: S12 Table — (DOCX) [file pone.0213531.s020.docx]

**Supplementary material**

**A study of CCD8 genes/proteins in seven monocots and eight dicots**

Ritu Batra^1^, Priyanka Agarwal^1^, Sandhya Tyagi^2^, Dinesh Kumar Saini^1^, Vikas Kumar^1^, Anuj Kumar^3^, Sanjay Kumar^4^, Harindra Singh Balyan^1^, Renu Pandey^2^

and Pushpendra Kumar Gupta^1^*

*Correspondence:

Pushpendra Kumar Gupta

email: [pkgupta36@gmail.com](mailto:pkgupta36@gmail.com)

**S12 Table.** Predicted scores of different parameters related to cellular component, biological processes and biochemical functions of CCD8 proteins of all the selected 15 species obtained through their functional analysis.

| Species | Cellular component | | | | Biological process | | | | Biochemical function | | | |
| --- | --- | --- | --- | --- | --- | --- | --- | --- | --- | --- | --- | --- |
|  | C | CP | I | IP | MP | OR | CP | PMP | CA | OA/a | OA/b | DA |
| *Z. mays* | 17.41 | 17.41 | 10.55 | 10.55 | 34.12 | 29.66 | 15.32 | 10.55 | 33.41 | 29.66 | 27.26 | 27.26 |
| *T.aestivum* sub-genome A | 13.9 | 13.9 | 11.76 | 11.76 | 40.99 | 36.17 | 13.83 | 13.08 | 40.51 | 36.17 | 26.87 | 26.87 |
| *T.aestivum* sub-genome B | 27.52 | 22.78 | 18.95 | 18.53 | 39.59 | 36.02 | 12.37 | 13.39 | 38.91 | 36.02 | 26.61 | 26.61 |
| *T.aestivum* sub-genome D | 8.71 | 8.71 | 5.98 | 5.19 | 25.16 | 18.95 | 10.36 | 8.87 | 27.52 | 22.78 | 18.95 | 18.53 |
| *T. urartu* | 10.76 | 10.76 | 9.07 | 9.07 | 41.19 | 31.62 | 15.3 | 17.32 | 37.6 | 31.62 | 30.08 | 30.81 |
| *A. tauschii* | 15.12 | 15.12 | 10.22 | 10.22 | 36.82 | 32.51 | 12.48 | 11.75 | 36.24 | 32.51 | 28.63 | 28.63 |
| *O. sativa* | 13.64 | 13.64 | 11.69 | 10.14 | 35.87 | 31.81 | 12.28 | 12.28 | 35.18 | 31.81 | 29.58 | 29.58 |
| *S. bicolor* | 15.52 | 15.52 | 9.03 | 9.03 | 37.02 | 33.41 | 12.35 | 10.86 | 34.06 | 32.68 | 28.11 | 28.11 |
| *B. distachyon* | 16.31 | 16.31 | 9.5 | 9.5 | 36.36 | 30.42 | 11.35 | 14.92 | 35.83 | 30.42 | 27.36 | 27.36 |
| *A. thaliana* | 21.82 | 21.82 | 15.4 | 15.4 | 46.19 | 29.57 | 19.62 | 17.07 | 45.15 | 31.46 | 31.46 | 27.41 |
| *S. lycopersicon* | 12.45 | 12.45 | 10.85 | 10.85 | 40.04 | 34.31 | 12.28 | 12.26 | 40.04 | 34.31 | 34.31 | 34.31 |
| *G. max* | 8.07 | 8.07 | 6.43 | 6.43 | 41.23 | 37.59 | 6.57 | 8.39 | 42.34 | 37.59 | 36.49 | 36.49 |
| *V. vinifera* | 12.29 | 12.29 | 5.39 | 6.19 | 40.82 | 37.91 | 10.91 | 7.25 | 39.13 | 37.91 | 35.49 | 35.49 |
| *P.persica* | 11.63 | 11.63 | 7.25 | 7.25 | 41.96 | 38.28 | 8.38 | 6.62 | 40.49 | 38.18 | 34.14 | 34.14 |
| *M.truncatula* | 10.07 | 10.07 | 6.44 | 7.33 | 23.97 | 19.37 | 7.41 | 8.05 | 22.72 | 17.09 | 19.37 | 14.92 |
| *T.cacao* | 7.83 | 7.83 | 6.94 | 6.94 | 40.09 | 35.2 | 7.82 | 8.65 | 40.03 | 35.2 | 34.53 | 34.53 |
| *P.trichocarpa* | 11.59 | 11.59 | 10.81 | 10.81 | 41.09 | 36.27 | 11.43 | 11.38 | 39.92 | 36.27 | 35.66 | 35.66 |

C-cell, CP-cell part, I-intracellular, IP-intracellular part; MP-metabolic process, OR-oxidation reduction, CP-cellular process, PMP-primary metabolic process, CA-catalytic activity, OA-oxidoreductase activity, DA-dioxygenase activity
